# Cardiometabolic risk factors in South American children: A systematic review and meta-analysis

**DOI:** 10.1371/journal.pone.0293865

**Published:** 2023-11-22

**Authors:** Carolyn M. H. Singleton, Sumeer Brar, Nicole Robertson, Lauren DiTommaso, George J. Fuchs, Aric Schadler, Aurelia Radulescu, Suzanna L. Attia

**Affiliations:** 1 University of Kentucky College of Medicine, Lexington, Kentucky, United States of America; 2 University of Kentucky College of Medicine Division of Pediatric Gastroenterology, Hepatology and Nutrition, Kentucky, United States of America; 3 University of Kentucky College of Public Health Department of Epidemiology, Kentucky, United States of America; 4 University of Kentucky College of Medicine Department of Pediatrics, Kentucky, United States of America; Gent University, BELGIUM

## Abstract

**Background:**

Cardiometabolic risk factors (impaired fasting glucose, abdominal obesity, high blood pressure, dyslipidemia) cluster in children, may predict adult disease burden, and are inadequately characterized in South American children.

**Objectives:**

To quantify the burden of cardiometabolic risk factors in South American children (0–21 years) and identify knowledge gaps.

**Methods:**

We systematically searched PubMed, Google Scholar, and the Latin American and Caribbean Health Sciences Literature via Virtual Health Library from 2000–2021 in any language. Two independent reviewers screened and extracted all data.

**Results:**

179 studies of 2,181 screened were included representing 10 countries (n = 2,975,261). 12.2% of South American children experienced obesity, 21.9% elevated waist circumference, 3.0% elevated fasting glucose, 18.1% high triglycerides, 29.6% low HDL cholesterol, and 8.6% high blood pressure. Cardiometabolic risk factor definitions varied widely. Chile exhibited the highest prevalence of obesity/overweight, low HDL, and impaired fasting glucose. Ecuador exhibited the highest prevalence of elevated blood pressure. Rural setting (vs. urban or mixed) and indigenous origin protected against most cardiometabolic risk factors.

**Conclusions:**

South American children experience high rates of obesity, overweight, and dyslipidemia. International consensus on cardiometabolic risk factor definitions for children will lead to improved diagnosis of cardiometabolic risk factors in this population, and future research should ensure inclusion of unreported countries and increased representation of indigenous populations.

## Introduction

Metabolic syndrome is the clustering of the cardiometabolic risk factors (CMRF) of impaired fasting glucose, abdominal obesity, high blood pressure (HBP), and dyslipidemia [1]. As of 2017, 20–25% of the world’s population was estimated to have metabolic syndrome (MetS) with an associated two or three times increased risk of death by heart attack or stroke respectively [1]. Childhood obesity is on the rise globally with an inconsistent understanding of the burden and impact of MetS in childhood and beyond [2]. Children with obesity are more likely to have obesity in adulthood, which is itself associated with significant health complications including gallstones, type 2 diabetes mellitus, non-alcoholic fatty liver disease, osteoarthritis, certain cancers, and cardiovascular events [3]. A rapid rise in obesity over the past fifty years is associated with a rise of 70% in healthcare treatment costs, and this now outweighs spending on non-communicable diseases associated with tobacco use or alcohol dependence [3].

### Defining cardiometabolic risk factors and metabolic syndrome in children

More than 40 different definitions of MetS in the pediatric population exist [4]. Two notable attempts to approach MetS definition in the pediatric population have been made by the International Diabetes Foundation (IDF) and the American Heart Association (AHA) [4]. The IDF definition states that the adult definition can be used down to the age of 10 years with the exception of a change in the definition of abdominal obesity, which is defined in children as ≥90th percentile for age and gender. The AHA did not explicitly create their own guidelines, but rather pointed to the conclusions of three studies, Cook et al, de Ferranti et al, and Ford et al, as appropriate starting points [4–7].

The rise in global obesity and CMRF is particularly relevant in South American countries, where cardiovascular disease is now the most common cause of death and disability [8]. Despite the increasing attention on CMRF globally and their short- and long-term effects both in populations and in an individual’s lifespan, the burden of CMRF in South American children is poorly defined [9]. Filling this knowledge gap could contribute to reducing this disease burden by illuminating the most common CMRF and bringing policy and healthcare attention to diagnosis, prevention, and treatment of at-risk children.

### Objective

The objective of this systematic review and meta-analysis was to quantify the burden of cardiometabolic risk factors in South American children, identify knowledge gaps, and propose next steps for research.

## Methods

### Inclusion/Exclusion criteria

**Inclusion Criteria:** We included all primary quantitative data from human subject studies on children ages 0–21 years from the geographical region of South America reported in any language. We included studies that reported prevalence data using any definition of at least one of the following: glucose intolerance; obesity; elevated waist circumference (WC); high blood pressure; and/or dyslipidemia, considered as low high-density lipoprotein (HDL), high low-density lipoprotein (LDL), and/or high triglyceride (TG). **Exclusion Criteria:** We excluded studies without available data, with data collected before 2000, focusing on a population with a chronic and/or congenital medical condition, duplicate data across multiple publications, duplicate data reported in different languages, and that did not report cutoff definitions for CMRF. We excluded the Caribbean islands due to their increased cultural and culinary heterogeneity compared to South America. Studies who recruited participants solely based on overweight/obese status were also excluded in order to minimize selection bias, as CMRF are higher in obese populations.

### Search strategy

**Fig 1:**
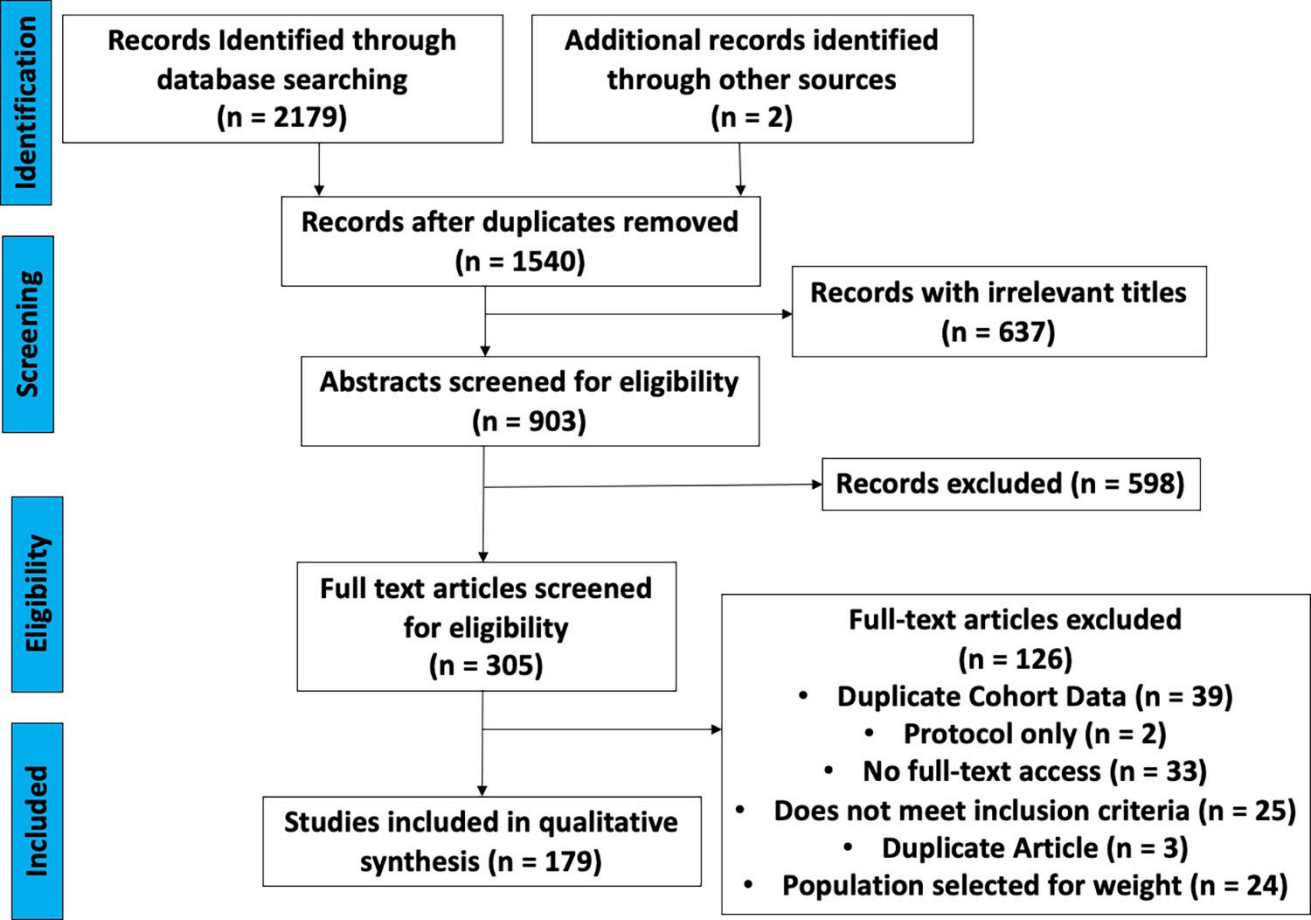
PRISMA flow chart.

Our search strategy is detailed in Table 1. We searched all available medical literature, including gray literature, using keyword searches in relevant databases. Our search strategy was composed of three main steps: 1) we performed an initial search of primary literature aggregators, ex. PubMed, to determine appropriate keyword terms in titles and abstracts; 2) we then used appropriate keywords to conduct a more thorough search of the literature; finally, 3) we examined references of articles found in step two to identify additional relevant data sources. We used Preferred Reporting Items for Systematic Review and Meta-analyses Protocols (PRISMA-P) as a framework to develop and guide our review [10]. An electronic search was performed using the databases PubMed, the Latin American and Caribbean Health Sciences Literature via Virtual Health Library, and Google Scholar. We examined the grey literature through the Google Scholar search. The initial search terms included: “*Cardiometabolic AND risk factor AND Latin America NOT adult*,*” “Cardiometabolic risk factor Ecuador*,*”* and *“Metabolic syndrome [MeSH Term] AND Latin America [MeSH Term]*.*”* MeSH terms were used when searching PubMed. The keywords around which the searches were developed included phrases revolving around CMRF, children, adolescents, and South America or Ecuador. We used Ecuador as the initial country to test and develop our search terms. This search strategy, specifically in Google Scholar, did not adequately represent all South American countries. Therefore, we expanded the Google Scholar search with advanced search features to combine all terms for each CMRF and individually searched all spelling variations for each South American country plus “children”. Fig 1 shows the PRISMA flow chart and screening process of all articles for the first search strategy.

**Table 1:** Search strategy.

Database	Search Terms	Hits
PubMed	(cardiometabolic risk children adolescents South America) OR (cardiometabolic risk factor and "Latin America"[Mesh])	74
Latin American and Caribbean Health Sciences Literature via Virtual Health Library	cardiometabolic risk factors children	43
Google Scholar Basic Search	cardiometabolic risk factors in Ecuadorian children	1,060*
Google Scholar Advanced Searches	allintitle: Argentina children obesity OR pressure OR systolic OR diastolic OR SBP OR DBP OR HBP OR HDL OR LDL OR TG OR hyperinsulinemia OR insulin OR "waist circumference" OR overweight OR anthropomorphic OR dyslipidemia OR glucose OR lipid OR cholesterol	21
	allintitle: Argentinian children obesity OR pressure OR systolic OR diastolic OR SBP OR DBP OR HBP OR HDL OR LDL OR TG OR hyperinsulinemia OR insulin OR "waist circumference" OR overweight OR anthropomorphic OR dyslipidemia OR glucose OR lipid OR cholesterol	5
	allintitle: Argentinean children obesity OR pressure OR systolic OR diastolic OR SBP OR DBP OR HBP OR HDL OR LDL OR TG OR hyperinsulinemia OR insulin OR "waist circumference" OR overweight OR anthropomorphic OR dyslipidemia OR glucose OR lipid OR cholesterol	22
	allintitle: Bolivia children obesity OR pressure OR systolic OR diastolic OR SBP OR DBP OR HBP OR HDL OR LDL OR TG OR hyperinsulinemia OR insulin OR "waist circumference" OR overweight OR anthropomorphic OR dyslipidemia OR glucose OR lipid OR cholesterol	3
	allintitle: Bolivian children obesity OR pressure OR systolic OR diastolic OR SBP OR DBP OR HBP OR HDL OR LDL OR TG OR hyperinsulinemia OR insulin OR "waist circumference" OR overweight OR anthropomorphic OR dyslipidemia OR glucose OR lipid OR cholesterol	4
	allintitle: Brazil children obesity OR pressure OR systolic OR diastolic OR SBP OR DBP OR HBP OR HDL OR LDL OR TG OR hyperinsulinemia OR insulin OR "waist circumference" OR overweight OR anthropomorphic OR dyslipidemia OR glucose OR lipid OR cholesterol	103
	allintitle: Brazilian children obesity OR pressure OR systolic OR diastolic OR SBP OR DBP OR HBP OR HDL OR LDL OR TG OR hyperinsulinemia OR insulin OR "waist circumference" OR overweight OR anthropomorphic OR dyslipidemia OR glucose OR lipid OR cholesterol	95
	allintitle: Brasil children obesity OR pressure OR systolic OR diastolic OR SBP OR DBP OR HBP OR HDL OR LDL OR TG OR hyperinsulinemia OR insulin OR "waist circumference" OR overweight OR anthropomorphic OR dyslipidemia OR glucose OR lipid OR cholesterol	7
	allintitle: Brasilian children obesity OR pressure OR systolic OR diastolic OR SBP OR DBP OR HBP OR HDL OR LDL OR TG OR hyperinsulinemia OR insulin OR "waist circumference" OR overweight OR anthropomorphic OR dyslipidemia OR glucose OR lipid OR cholesterol	0
	allintitle: Chile children obesity OR pressure OR systolic OR diastolic OR SBP OR DBP OR HBP OR HDL OR LDL OR TG OR hyperinsulinemia OR insulin OR "waist circumference" OR overweight OR anthropomorphic OR dyslipidemia OR glucose OR lipid OR cholesterol	30
	allintitle: Chilean children obesity OR pressure OR systolic OR diastolic OR SBP OR DBP OR HBP OR HDL OR LDL OR TG OR hyperinsulinemia OR insulin OR "waist circumference" OR overweight OR anthropomorphic OR dyslipidemia OR glucose OR lipid OR cholesterol	65
	allintitle: Colombia children obesity OR pressure OR systolic OR diastolic OR SBP OR DBP OR HBP OR HDL OR LDL OR TG OR hyperinsulinemia OR insulin OR "waist circumference" OR overweight OR anthropomorphic OR dyslipidemia OR glucose OR lipid OR cholesterol	21
	allintitle: Colombian children obesity OR pressure OR systolic OR diastolic OR SBP OR DBP OR HBP OR HDL OR LDL OR TG OR hyperinsulinemia OR insulin OR "waist circumference" OR overweight OR anthropomorphic OR dyslipidemia OR glucose OR lipid OR cholesterol	23
	allintitle: Ecuador children obesity OR pressure OR systolic OR diastolic OR SBP OR DBP OR HBP OR HDL OR LDL OR TG OR hyperinsulinemia OR insulin OR "waist circumference" OR overweight OR anthropomorphic OR dyslipidemia OR glucose OR lipid OR cholesterol	9
	allintitle: Ecuadorian children obesity OR pressure OR systolic OR diastolic OR SBP OR DBP OR HBP OR HDL OR LDL OR TG OR hyperinsulinemia OR insulin OR "waist circumference" OR overweight OR anthropomorphic OR dyslipidemia OR glucose OR lipid OR cholestero	3
	allintitle: Paraguay children obesity OR pressure OR systolic OR diastolic OR SBP OR DBP OR HBP OR HDL OR LDL OR TG OR hyperinsulinemia OR insulin OR "waist circumference" OR overweight OR anthropomorphic OR dyslipidemia OR glucose OR lipid OR cholesterol	0
	allintitle: Paraguayan children obesity OR pressure OR systolic OR diastolic OR SBP OR DBP OR HBP OR HDL OR LDL OR TG OR hyperinsulinemia OR insulin OR "waist circumference" OR overweight OR anthropomorphic OR dyslipidemia OR glucose OR lipid OR cholesterol	0
	allintitle: Peru children obesity OR pressure OR systolic OR diastolic OR SBP OR DBP OR HBP OR HDL OR LDL OR TG OR hyperinsulinemia OR insulin OR "waist circumference" OR overweight OR anthropomorphic OR dyslipidemia OR glucose OR lipid OR cholesterol	15
	allintitle: Peruvian children obesity OR pressure OR systolic OR diastolic OR SBP OR DBP OR HBP OR HDL OR LDL OR TG OR hyperinsulinemia OR insulin OR "waist circumference" OR overweight OR anthropomorphic OR dyslipidemia OR glucose OR lipid OR cholesterol	9
	allintitle: Suriname children obesity OR pressure OR systolic OR diastolic OR SBP OR DBP OR HBP OR HDL OR LDL OR TG OR hyperinsulinemia OR insulin OR "waist circumference" OR overweight OR anthropomorphic OR dyslipidemia OR glucose OR lipid OR cholesterol	2
	allintitle: Surinamese children obesity OR pressure OR systolic OR diastolic OR SBP OR DBP OR HBP OR HDL OR LDL OR TG OR hyperinsulinemia OR insulin OR "waist circumference" OR overweight OR anthropomorphic OR dyslipidemia OR glucose OR lipid OR cholesterol	4
	allintitle: Uruguay children obesity OR pressure OR systolic OR diastolic OR SBP OR DBP OR HBP OR HDL OR LDL OR TG OR hyperinsulinemia OR insulin OR "waist circumference" OR overweight OR anthropomorphic OR dyslipidemia OR glucose OR lipid OR cholesterol	1
	allintitle: Uruguayan children obesity OR pressure OR systolic OR diastolic OR SBP OR DBP OR HBP OR HDL OR LDL OR TG OR hyperinsulinemia OR insulin OR "waist circumference" OR overweight OR anthropomorphic OR dyslipidemia OR glucose OR lipid OR cholesterol	2
	allintitle: Venezuela children obesity OR pressure OR systolic OR diastolic OR SBP OR DBP OR HBP OR HDL OR LDL OR TG OR hyperinsulinemia OR insulin OR "waist circumference" OR overweight OR anthropomorphic OR dyslipidemia OR glucose OR lipid OR cholesterol	11
	allintitle: Venezuelan children obesity OR pressure OR systolic OR diastolic OR SBP OR DBP OR HBP OR HDL OR LDL OR TG OR hyperinsulinemia OR insulin OR "waist circumference" OR overweight OR anthropomorphic OR dyslipidemia OR glucose OR lipid OR cholesterol	2
	allintitle: Falkland island children obesity OR pressure OR systolic OR diastolic OR SBP OR DBP OR HBP OR HDL OR LDL OR TG OR hyperinsulinemia OR insulin OR "waist circumference" OR overweight OR anthropomorphic OR dyslipidemia OR glucose OR lipid OR cholesterol	0
	allintitle: French Guiana children obesity OR pressure OR systolic OR diastolic OR SBP OR DBP OR HBP OR HDL OR LDL OR TG OR hyperinsulinemia OR insulin OR "waist circumference" OR overweight OR anthropomorphic OR dyslipidemia OR glucose OR lipid OR cholesterol	0

*Due to a Google Scholar server error, only 980 of the 1060 results were imported.

PRISMA 2009 flow diagram. From: Moher D, Liberati A, Tetzlaff J, Altman DG, The PRISMA Group (2009). Preferred Reporting Items for Systematic Reviews and Meta-Analyses: The PRISMA Statement. PLoS Med [10].

### Study selection and data extraction

One reviewer (CH) screened titles and abstracts of the retrieved articles for relevance, and a second reviewer (SB) independently confirmed relevance. Disputes were settled by an arbitrator (SLA). Fig 1 shows the associated PRISMA flowchart of study selection. Two independent people (CH, SB, LD, and/or NR) performed data extraction and assessment of study quality. After comparison of extracted data, disputes were settled through an arbitrator (SLA). For studies that included an intervention, only baseline data was recorded so that interventions could not skew initial population prevalence of CMRF.

### Outcomes

Primary outcomes were prevalence of CMRF and study quality. CMRF definitions used by each study were also recorded. Due to the high amount of heterogeneity in the definitions used, we completed separate analyses with the most common definition for each CMRF to reduce heterogeneity as much as possible. Secondary outcomes were demographic data and study characteristics including age; study setting: rural, mixed, or urban; inclusion of indigenous population; year(s) of data collection; gender distribution: male or female; study location; study design; and study inclusion and exclusion criteria.

### Study quality assessment

Study quality was assessed using a modified Effective Public Healthcare Panacea Project Quality Assessment Tool for Quantitative Studies [11]. Studies were graded on selection bias and data collection methods, then graded as either “Strong,” “Moderate,” or “Weak” based on their pooled scores. Quality ratings for included titles are presented in Table 2.

**Table 2:** Included studies assessing cardiometabolic risk factors in South American children.

Author (et al), year	Years of data collection	Country	City/Region	Study Setting	Risk Factor(s) Studied	Selection Bias Rating	Data Collection Methods Rating	Overall Rating
**Abril, 2013 [12]**	2010–2011	Ecuador	Cuenca	Urban	Obesity, WC	Strong	Strong	Strong
**Aglony, 2009 [13]**	2005–2006	Chile	Santiago	Urban	Obesity, HBP	Moderate	Strong	Strong
**Aguilar Salinas, 2016 [14]**	2015	Ecuador	Quito and Cariamanga	Mixed	Obesity, WC	Moderate	Strong	Strong
**Albuquerque, 2018 [15]**	2016	Brazil	Viçosa	Urban	Obesity, WC, GI, Dyslipidemia, HBP	Moderate	Moderate	Moderate
**Alexius, 2012 [16]**	Not Reported	Brazil	Medianeira	Mixed	Obesity	Strong	Strong	Strong
**Alves, 2009 [17]**	2006	Brazil	Recife	Urban	Obesity	Strong	Moderate	Strong
**Andaki, 2018 [18]**	Not Reported	Brazil	Uberaba	Mixed	Obesity, GI, Dyslipidemia, HBP	Moderate	Strong	Strong
**Andrade, 2014 [19]**	2008–2009	Ecuador	Cuenca and Nabon	Mixed	Dyslipidemia	Strong	Strong	Strong
**Andrade de Medieros Moreira, 2020 [20]**	2018	Brazil	Palmas	Urban	Obesity	Moderate	Moderate	Moderate
**Araujo, 2017 [21]**	2012–2013	Brazil	Piracicaba	Urban	Obesity	Moderate	Weak	Moderate
**Araujo, 2016 [22]**	Not Reported	Brazil	São Caetano do Sul	Urban	Obesity	Moderate	Weak	Moderate
**Arias Téllez, 2018 [23]**	2008–2011	Chile	Santiago	Urban	Obesity, WC	Weak	Moderate	Moderate
**Aristizábal, 2019 [24]**	2015	Colombia	Medellín	Mixed	Obesity, WC	Weak	Strong	Moderate
**Assis, 2006 [25]**	2002	Brazil	Florianópolis	Urban	Obesity	Moderate	Strong	Strong
**Assunçáo, 2015 [26]**	2005	Brazil	multiple states	Mixed	Obesity	Moderate	Moderate	Moderate
**Barbalho, 2017 [27]**	Not Reported	Brazil	Lins	Mixed	Obesity, WC, GI, Dyslipidemia	Weak	Strong	Strong
**Barja, 2011 [28]**	2009–2010	Chile	Puente Alto	Urban	Obesity, WC, GI	Moderate	Strong	Strong
**Barros Costa, 2003 [29]**	2000	Brazil	Juiz de Fora	Mixed	Obesity	Moderate	Moderate	Moderate
**Bauce, 2018 [30]**	2010–2011	Venezuela	Caracas	Urban	Obesity	Moderate	Moderate	Moderate
Bénéfice*, 2007 [31]	2004–2005	Bolivia	Beni River/ Amazon	Rural	Obesity	Moderate	Strong	Strong
**Benini, 2017 [32]**	2011–2012	Brazil	Garibaldi	Urban	Obesity, WC, GI, Dyslipidemia	Moderate	Strong	Strong
**Berghtein, 2014 [33]**	2012	Argentina	Río Grande, Tierra del Fuego	Mixed	Obesity	Strong	Moderate	Strong
**Berria, 2013 [34]**	2006	Brazil	Cascavel	Urban	Obesity, WC	Strong	Moderate	Strong
**Brinkman, 2015 [35]**	2013	Chile	Punta Arenas	Urban	Obesity	Strong	Moderate	Strong
**Bozzini, 2019 [36]**	2014–2017	Brazil	São Paulo	Urban	Obesity, Dyslipidemia	Moderate	Moderate	Moderate
**Buitrago-Lopez, 2015 [37]**	2006–2007	Colombia	Bucaramanga	Urban	Obesity	Moderate	Strong	Strong
**Burgos, 2019 [38]**	2011–2012	Brazil	Santa Cruz do Sul	Mixed	Obesity, WC, Dyslipidemia, HBP	Strong	Weak	Moderate
**Burrows, 2015 [39]**	Not Reported	Chile	Santiago	Urban	Obesity, WC, GI, Dyslipidemia, HBP	Weak	Strong	Moderate
**Bustamante, 2013 [40]**	2009–2010	Peru	Central region	Mixed	Obesity, WC	Strong	Moderate	Strong
**Caamaño Navarrete, 2015 [41]**	Not Reported	Chile	Temuco	Urban	Obesity	Moderate	Moderate	Strong
**Caixeta, 2020 [42]**	2016–2017	Brazil	Brasilia	Urban	Obesity, WC	Strong	Moderate	Strong
**Calvo, 2004 [43]**	2002	Brazil	Florianopolis	Urban	Obesity	Strong	Moderate	Strong
**Campos, 2006 [44]**	2003	Brazil	Fortaleza	Urban	Obesity	Moderate	Moderate	Moderate
**Campoverde Ríos, 2016 [45]**	Not Reported	Ecuador	Quito	Urban	Obesity, WC, GI	Moderate	Moderate	Moderate
**Carolina Avalos, 2012 [46]**	2006–2007	Chile	Santiago	Urban	Obesity	Moderate	Strong	Strong
**Carrillo-Larco, 2016 [47]**	2006–2007	Peru		Mixed	Obesity	Moderate	Moderate	Moderate
**Casagrande, 2017 [48]**	2015	Brazil	Marília	Mixed	Obesity, WC	Moderate	Strong	Strong
**Casapulla, 2017 [49]**	2015	Ecuador	Pomasqui and Cariamanga	Urban	Obesity, GI	Moderate	Strong	Strong
**Castro Burbano, 2016 [50]**	Not Reported	Ecuador		Mixed	Obesity	Moderate	Moderate	Moderate
**Cediel, 2016 [51]**	2002- ongoing	Chile	Santiago	Urban	Obesity, WC, GI	Moderate	Strong	Strong
**Cesani, 2020 [52]**	2015–2017	Artentina	La Plata	Mixed	Obesity, HBP	Strong	Strong	Strong
**Cobo, 2015 [53]**	2011	Chile	Quillota	Mixed	Obesity, HBP	Moderate	Moderate	Moderate
**Cohen, 2016 [54]**	2012	Argentina	Pilar Buenos Aires	Mixed	Obesity, HBP	Moderate	Strong	Strong
**Cohen, 2014 [55]**	2011–2012	Colombia	Bucaramanga	Urban	Obesity	Moderate	Moderate	Moderate
**Collazo, 2018 [56]**	2016–2017	Ecuador	Cuenca	Mixed	Obesity	Moderate	Moderate	Moderate
**Corso, 2004 [57]**	2002	Brazil	Floranópolis	Mixed	Obesity	Moderate	Moderate	Moderate
**Corvalán, 2013 [58]**	2002- ongoing	Chile	Santiago	Urban	Obesity, WC	Strong	Strong	Strong
**Corvalan, 2010 [59]**	2006	Chile	Santiago	Urban	Obesity, WC, GI, Dyslipidemia	Moderate	Strong	Strong
**Costa, 2006 [60]**	2002	Brazil	Santos	Urban	Obesity	Strong	Moderate	Strong
**Crovetto, 2010 [61]**	2008	Chile	Valparíso	Urban	Obesity	Strong	Weak	Moderate
**Cruz, 2017 [62]**	2008	Brazil	Pelotas	Urban	Obesity	Moderate	Weak	Moderate
**da Silva, 2013 [63]**	2005–2006	Brazil	Alagotas State	Mixed	Obesity	Moderate	Strong	Strong
**de Carvahlo Cremm, 2011 [64]**	Not Reported	Brazil	Santos	Urban	Obesity	Moderate	Moderate	Moderate
**de Melo, 2016 [65]**	2010–2012	Brazil	Natal	Urban	Obesity, WC, GI, Dyslipidemia, HBP	Moderate	Moderate	Moderate
**Delgado-Floody, 2019 [66]**	Not Reported	Chile	Araucania Region	Mixed	Obesity, HBP	Moderate	Moderate	Moderate
**Delgado-Floody, 2017 [67]**	Not Reported	Chile		Mixed	Obesity, WC	Moderate	Strong	Strong
**De Santis Filgueiras, 2018 [68]**	2015	Brazil	Viçosa, Minas Gerais	Mixed	Obesity, WC	Strong	Strong	Strong
**De Santis Filgueiras, 2018 [69]**	2015	Brazil	Viçosa, Minas Gerais	Mixed	Dyslipidemia	Strong	Strong	Strong
**Devia Solia, 2018 [70]**	2017–2018	Ecuador	Azogues	Urban	Obesity, WC, Dyslipidemia, HBP	Weak	Strong	Moderate
**Diaz, 2010 [71]**	Not Reported	Argentina	Maria Ignacia Vela	Rural	Obesity, HBP	Moderate	Moderate	Moderate
**Duncan, 2011 [72]**	Not Reported	Brazil	São Paulo state	Mixed	Obesity	Strong	Moderate	Strong
**Escalona-Villasmil, 2016 [73]**	2013	Venezuela	Marcaibo	Urban	Obesity	Weak	Strong	Moderate
**Ferrari, 2019 [74]**	Not Reported	Brazil	São Caetano do Sul	Urban	Obesity	Moderate	Strong	Strong
**Ferreira, 2009 [75]**	Not Reported	Brazil	Taguatinga, Brasília	Mixed	Obesity	Moderate	Strong	Strong
**Figueroa Sobero, 2016 [76]**	Not Reported	Argentina	Riós, Buenos Aires, CABC, Salta, Córdoba, Tierra del Fuego, Corrientes	Urban	Obesity, WC, GI, Dyslipidemia, HBP	Moderate	Strong	Strong
**Florencio de Souza, 2011 [77]**	2004	Brazil	Rio Branco, Acre State	Mixed	Obesity	Strong	Moderate	Strong
**Fornasini, 2016 [78]**	Not Reported	Ecuador	Quito	Urban	Obesity	Moderate	Weak	Moderate
**Gamboa-Delgado, 2017 [79]**	2006–2007	Colombia	Bucaramanga	Urban	Obesity, GI, Dyslipidemia, HBP	Moderate	Strong	Strong
**Game, 2015 [80]**	Not Reported	Chile	Boyeco	Urban	Obesity	Moderate	Weak	Moderate
**Garcia-Hermoso, 2019 [81]**	2016–2017	Chile	Santiago	Urban	Obesity, HBP	Moderate	Moderate	Moderate
**Gaya, 2017 [82]**	2008–2009 and 2013–2014	Brazil	16 states	Mixed	Obesity	Moderate	Strong	Strong
**Gilbert-Diamond, 2012 [83]**	2006	Colombia	Bogotá	Urban	Obesity	Strong	Strong	Strong
**Giuliano, 2011 [84]**	2002–2004	Brazil	Florianópolis	Urban	Dyslipidemia	Moderate	Moderate	Strong
**Gómes, 2019 [85]**	2008–2015	Brazil	Campinas	Mixed	Dyslipidemia	Strong	Strong	Strong
**Gomes, 2017 [86]**	2004–2005	Brazil	Feria de Santana	Mixed	Obesity	Moderate	Strong	Strong
**Gomez, 2007 [87]**	2005	Colombia		Mixed	Obesity	Moderate	Moderate	Moderate
**Granville-Garcia, 2008 [88]**	Not Reported	Brazil	Recife	Urban	Obesity	Moderate	Moderate	Moderate
**Guedes, 2013 [89]**	2011	Brazil	Monte Claros	Mixed	Obesity	Strong	Strong	Strong
**Guevara, 2018 [90]**	2015–2016	Ecuador	Riobamba	Urban	Obesity	Moderate	Weak	Moderate
**Gutierez-Gomez, 2009 [91]**	2006	Chile		Mixed	Obesity, WC	Strong	Strong	Strong
**Hércules, 2020 [92]**	2016–2017	Brazil	Curibata	Mixed	Obesity	Moderate	Moderate	Moderate
**Herrán, 2017 [93]**	2009–2011	Colombia		Mixed	Obesity	Moderate	Moderate	Moderate
**Herrera Sevilla, 2015 [94]**	2015	Ecuador	Quito	Urban	Obesity	Weak	Weak	Weak
Hirshler*, 2014 [95]	2011–2013	Argentina	San Antonio de los Cobres	Rural	Obesity	Moderate	Moderate	Moderate
Hirschler*, 2012 [96]	2007–2008	Argentina	San Antonio de los Cobres and Buenos Aires	Mixed	Obesity, WC, GI, Dyslipidemia, HBP	Moderate	Strong	Strong
**Hirschler, 2009 [97]**	2007	Argentina	Buenos Aires	Urban	Obesity	Moderate	Moderate	Moderate
**Hirschler, 2010 [98]**	2007	Argentina	Buenos Aires	Urban	Obesity, WC, GI, Dyslipidemia, HBP	Strong	Strong	Strong
Hirschler*, 2016 [99]	2011–2014	Argentina	San Antonio de los Cobres	Rural	Obesity, Dyslipidemia	Strong	Moderate	Strong
**Hirschler, 2006 [100]**	2004	Argentina	Buenos Aires	Urban	Obesity	Moderate	Moderate	Moderate
Hirschler*, 2015 [101]	2012–2013	Argentina	San Antonio de los Cobres and Buenos Aires	Mixed	Obesity, Dyslipidemia	Strong	Moderate	Strong
Hirschler*, 2013 [102]	Not Reported	Argentina	San Antonio de los Cobres	Rural	Obesity	Strong	Strong	Strong
**Honório, 2014 [103]**	2011–2012	Brazil	Goiânia	Urban	Obesity	Moderate	Strong	Strong
**Houck, 2017 [104]**	Not Reported	Ecuador	Galapagos	Mixed	Obesity	Moderate	Weak	Moderate
**Houck, 2013 [105]**	2009	Ecuador	Northern Ecuador Amazon	Rural	Obesity	Moderate	Strong	Moderate
Iguarán Kohen*, 2015 [106]	2015	Colombia	Siapana	Rural	Obesity, WC	Moderate	Strong	Strong
**Inciarte, 2013 [107]**	Not Reported	Venezuela	Maracaibo	Urban	Obesity	Moderate	Moderate	Moderate
**Jones, 2018 [108]**	2015	Bolivia	El Alto and Montero	Mixed	Obesity	Moderate	Strong	Strong
**Kain, 2014 [109]**	2011–2012	Chile	Nuñoa	Mixed	Obesity	Strong	Weak	Moderate
**Kain, 2007 [110]**	2002–2004	Chile	Santiago	Mixed	Obesity	Moderate	Weak	Moderate
**Kain, 2016 [111]**	2006 onward	Chile		Mixed	Obesity, WC	Moderate	Moderate	Moderate
**Kain, 2009 [112]**	2006	Chile	Santiago	Urban	Obesity	Strong	Strong	Strong
**Kain, 2009 [113]**	2003	Chile	Casablanca and Quillota	Mixed	Obesity	Moderate	Strong	Strong
**Kupek, 2014 [114]**	2007	Brazil	Florianopolis	Mixed	Obesity	Moderate	Moderate	Moderate
**Leal, 2012 [115]**	2006	Brazil	Pernambuco State	Mixed	Obesity	Strong	Moderate	Strong
**Leite, 2017 [116]**	2001–2014	Brazil	Juiz de Fora	Mixed	Obesity	Moderate	Weak	Moderate
**Linares Herrera, 2017 [117]**	2015	Peru	Tarapoto	Urban	Obesity, WC	Moderate	Strong	Strong
**Lizana, 2016 [118]**	2013–2014	Chile	Quilota and Valparaíso	Mixed	Obesity, WC	Moderate	Weak	Moderate
**Loaiza, 2012 [119]**	Not Reported	Chile		Mixed	Obesity	Moderate	Weak	Moderate
**Lourenço, 2014 [120]**	2007–2009	Brazil	Acrelândia	Mixed	Obesity	Moderate	Strong	Strong
**Matos, 2011 [121]**	2005	Brazil	Salvador	Urban	Obesity, GI	Moderate	Strong	Strong
**Morillo Silva, 2017 [122]**	Not Reported	Ecuador	Santo Domingo	Urban	Obesity	Weak	Weak	Weak
**Muñoz, 2016 [123]**	Not Reported	Colombia	Medellín	Urban	Obesity, WC, GI, Dyslipidemia, HBP	Moderate	Strong	Strong
**Musso, 2011 [124]**	2008	Argentina	Buenos Aires	Urban	Obesity, WC, GI, Dyslipidemia, HBP	Moderate	Strong	Strong
**Naves, 2006 [125]**	2005	Brazil	Brasilia	Urban	Dyslipidemia	Moderate	Strong	Strong
**Neyla de Lima Albaquerque, 2016 [126]**	2013	Brazil	Recife	Urban	Obesity, WC, Dyslipidemia	Weak	Strong	Moderate
**Ninatana-Ortiz, 2016 [127]**	2014	Peru	Cajamarca	Mixed	Obesity, WC, GI, Dyslipidemia, HBP	Moderate	Strong	Strong
**Novaes, 2011 [128]**	2005–2006	Brazil	Visçosa	Mixed	Obesity	Moderate	Moderate	Moderate
**Obregón, 2018 [129]**	2014–2015	Chile	Concepción	Mixed	Obesity	Strong	Moderate	Moderate
**Olaya-Contreras, 2015 [130]**	2013	Colombia	Medellin	Urban	Obesity	Moderate	Moderate	Strong
**Oliveira Pani, 2015 [131]**	Not Reported	Brazil	Espiritu Santo province	Urban	Obesity, Dyslipidemia	Weak	Weak	Weak
**Oliveira, 2007 [132]**	Not Reported	Brazil	Feria de Santana	Urban	Obesity	Moderate	Moderate	Moderate
**Orden, 2018 [133]**	2015–2016	Argentina	Santa Rosa	Urban	Obesity	Moderate	Strong	Strong
**Padula, 2012 [134]**	2003–2005	Argentina	La Plata, Buenos Aires	Urban	Obesity	Moderate	Strong	Strong
**Pajuelo-Ramírez, 2013 [135]**	2009–2010	Peru		Mixed	Obesity	Moderate	Moderate	Moderate
**Pajuelo-Ramírez, 2011 [136]**	2007–2010	Peru		Mixed	Obesity	Moderate	Moderate	Moderate
**Passos, 2015 [137]**	2012	Brazil	Pelotas	Mixed	Obesity	Moderate	Strong	Strong
**Peregalli, 2013 [138]**	Not Reported	Uruguay		Mixed	Obesity	Moderate	Weak	Moderate
**Pereira, 2009 [139]**	2001	Brazil	Itapetininga	Mixed	Obesity, Dyslipidemia, HBP	Moderate	Moderate	Moderate
**Pereira, 2013 [140]**	2004–2006	Brazil	Jundiai	Urban	Obesity, Dyslipidemia	Moderate	Weak	Moderate
**Pereyra, 2021 [141]**	2013–2014 and 2015–2016	Uruguay		Mixed	Obesity	Moderate	Weak	Moderate
**Pincón, 2011 [142]**	2010–2011	Ecuador	Cuenca	Rural	Obesity	Weak	Moderate	Moderate
**Poveda, 2007 [143]**	Not Reported	Colombia		Mixed	Obesity	Moderate	Moderate	Moderate
**Quadros, 2016 [144]**	2011–2012	Brazil	Amargosa	Mixed	Obesity, WC, GI, Dyslipidemia, HBP	Moderate	Moderate	Moderate
**Ramirez-Velez, 2017 [145]**	2013–2016	Colombia	Bogota	Urban	Obesity, WC, GI, Dyslipidemia, HBP	Moderate	Strong	Strong
**Ramirez-Velez, 2016 [146]**	2013–2016	Colombia	Bogotá	Urban	Obesity	Moderate	Strong	Strong
**Ramos-Padilla, 2015 [147]**	2013	Ecuador	Riobamba	Urban	Obesity	Moderate	0	Moderate
**Real Delor, 2017 [148]**	2020	Paraguay	Asunción	Urban	Obesity, HBP	Moderate	Moderate	Moderate
**Ribas, 2012 [149]**	2005	Brazil	Belém	Urban	Obesity, WC, Dyslipidemia	Moderate	Weak	Moderate
**Ribeiro, 2017 [150]**	Not Reported	Brazil	São Luís	Urban	Obesity	Moderate	Strong	Strong
**Ricardo, 2009 [151]**	2007–2008	Brazil	Santa Catarina state	Mixed	Obesity	Strong	Strong	Strong
**Rinaldi, 2010 [152]**	2007	Brazil	Botucatu	Urban	Obesity	Moderate	Strong	Strong
**Rincón, 2015 [153]**	2010–2011	Vanezuela	Merida	Urban	Obesity, GI, Dyslipidemia, HBP	Strong	Strong	Strong
**Rizzo, 2013 [154]**	2009–2011	Brazil	Botucatu	Urban	Obesity, WC, GI, Dyslipidemia, HBP	Moderate	Moderate	Moderate
**Rodrigues-Bezerra, 2016 [155]**	2014–2015	Colombia	Bogotá	Urban	Obesity	Strong	Strong	Strong
**Romagna, 2010 [156]**	2007–2008	Brazil	Canoes	Mixed	Obesity	Moderate	Strong	Strong
**Romero-Sandoval, 2012 [157]**	2010–2011	Ecuador	Quito	Urban	Obesity	Strong	Moderate	Strong
**Rosini, 2013 [158]**	2009	Brazil	Guabiruba	Mixed	Obesity, WC	Weak	Strong	Moderate
**Rossi, 2018 [159]**	Not Reported	Ecuador	Galápagos	Urban	Obesity	Moderate	Moderate	Moderate
**Ruiz, 2014 [160]**	2012–2013	Venezuela	Valencia	Urban	Obesity, WC, GI	Weak	Moderate	Moderate
**Salazar-Guitérrez, 2020 [161]**	Not Reported	Chile	Chillán	Mixed	Obesity	Moderate	Moderate	Moderate
**Salceda, 2013 [162]**	2004	Argentina	Catamarca	Mixed	Obesity	Moderate	Moderate	Moderate
**Saldiva, 2004 [163]**	2001	Brazil	Bady Bassit, Bofete, Jaborandi, Morungaba, Riversul	Mixed	Obesity	Strong	Strong	Strong
**Saldiva, 2007 [164]**	2001	Brazil	Bady Bassit, Bofete, Jaborandi, Morungaba, Riversul	Mixed	Obesity	Moderate	Strong	Strong
**Santos, 2019 [165]**	2009–2010	Peru	Barranco, La Merced, San Ranom, Junín	Mixed	Obesity	Moderate	Moderate	Moderate
**Sapunar, 2018 [166]**	2015–2016	Chile	Carahue	Urban	Obesity, WC, GI, Dyslipidemia, HBP	Moderate	Weak	Moderate
**Sehn, 2016 [167]**	Not Reported	Brazil	Santa Cruz do Sul	Urban	Obesity	Moderate	Weak	Moderate
**Sentalin, 2019 [168]**	2015	Brazil	Vinhedo	Mixed	Obesity, WC	Moderate	Moderate	Moderate
**Serrano, 2019 [169]**	2006–2017	Colombia	Bucaramanga	Urban	Obesity, WC, GI, Dyslipidemia, HBP	Weak	Strong	Moderate
**Silva, 2018 [170]**	2012–2013	Brazil	Uberaba	Mixed	Obesity	Moderate	Moderate	Moderate
**Silva, 2011 [171]**	2008–2010	Brazil	Sergipe	Mixed	Obesity	Moderate	Weak	Moderate
**Silva, 2009 [172]**	2005	Brazil	João Pessoa	Mixed	HBP	Strong	Strong	Strong
**Silveira, 2014 [173]**	2006–2007	Brazil		Mixed	Obesity	Moderate	Moderate	Moderate
**Solano, 2003 [174]**	2002	Venezuela	Valencia	Urban	Obesity, Dyslipidemia	Moderate	Weak	Moderate
**Souza, 2021 [175]**	2016	Brazil	Monte Negro	Mixed	Obesity, Dyslipidemia, HBP	Moderate	Strong	Strong
**Suarez-Lopez, 2019 [176]**	2008	Ecuador	Pedro Moncayo County	Rural	HBP	Moderate	Strong	Strong
**Suarez-Ortegón, 2016 [177]**	Not Reported	Colombia	Cali	Urban	Obesity, WC, GI, Dyslipidemia, HBP	Moderate	Strong	Strong
**Tande da Silva, 2011 [178]**	2007–2009	Brazil	São Paulo	Urban	Obesity	Weak	Strong	Moderate
**Tardivo, 2013 [179]**	Not Reported	Brazil	Sao Paulo	Mixed	Obesity	Moderate	Weak	Moderate
**Tarqui-Mamani, 2018 [180]**	2013–2014	Peru		Mixed	Obesity	Moderate	Moderate	Moderate
**Todendi, 2019 [181]**	2014–2015	Brazil	Santa Cruz do sul	Mixed	Obesity, WC	Moderate	Moderate	Moderate
**Torres-Roman, 2018 [182]**	2010–2015	Peru		Mixed	Obesity	Moderate	Moderate	Moderate
**Torres, 2015 [183]**	2010–2011	Brazil	Maruípe, Continebtal	Mixed	Obesity	Moderate	Weak	Moderate
**Toso, 2003 [184]**	Not Reported	Brazil	Jaragua do sul	Mixed	Obesity	Moderate	Weak	Moderate
**Ulloa, 2010 [185]**	2006	Chile	Concepción, Coronel, Hualpén	Urban	Obesity	Strong	Weak	Moderate
**Vanzelli, 2008 [186]**	2005	Brazil	Jundiaí	Urban	Obesity	Moderate	Strong	Strong
**Verona, 2013 [187]**	Not Reported	Argentina	Balcarce	Rural	Obesity, WC, GI, Dyslipidemia, HBP	Moderate	Strong	Strong
**Villalba-Condori, 2019 [188]**	Not Reported	Peru		Mixed	Obesity	Weak	Weak	Weak
**Zaccarelli-Marino, 2020 [189]**	2011–2012	Brazil	Santo André	Urban	Obesity	Moderate	Moderate	Moderate
**Zandoná, 2017 [190]**	2001–2005, 2008	Brazil	Leopoldo and Porto Alegre	Mixed	Obesity	Moderate	Moderate	Moderate

*indicates study included indigenous population; WC = waist circumference; GI = glucose intolerance; HBP = high blood pressure.

### Statistical analysis

We performed descriptive statistics with Microsoft® Excel for Mac Version 16.54 and descriptive and analytical statistics with SPSS 28. Due to the risk of confounding variables and the influence of study heterogeneity, we performed a summative sub-analysis of median prevalence rates of CMRF using the same definition. We used nonparametric, univariate, and multivariate testing. For variables with two groups, we utilized the Independent-Samples Mann-Whitney U Test, and for variables with more than two groups we used the Independent-Samples Kruskal-Wallis Test.

## Results

### Study characteristics and population demographics

Of 2,181 studies screened, we included 179 studies. These were comprised of 2,975,261 unique subjects representing ten countries (Argentina, Bolivia, Brazil, Chile, Colombia, Ecuador, Paraguay, Peru, Uruguay, and Venezuela). Our search did not find relevant results for the following countries and territories in South America: Guyana, Suriname, French Guiana, and the Falkland Islands. Peru had the largest population with 2,380,473 subjects, followed by Brazil with 276,648. Paraguay had the least with 132 subjects. The range of female subjects was 0–100% with median of 51.0%. Eight studies (4.5%) included indigenous populations. Study settings were 6.2% rural, 48.0% urban, and 45.8% mixed settings.

### Cardiometabolic risk factor definitions

The definitions for each risk factor are presented in Table 3. Included studies used 15 different definitions for obesity, 8 definitions for elevated waist circumference, 4 definitions for impaired fasting glucose, 11 definitions for elevated triglycerides, 9 definitions for low HDL cholesterol, and 8 definitions for HBP.

**Table 3:** All definitions used for cardiometabolic risk factors in studies assessing South American Children.

Risk Factor	Definitions Used (n = number of studies used definition)
**Obesity (159)**	BMI, WH, WL ≥95% for age and gender (53, 33.3%); BMIZ ≥ +2 above mean (45, 28.3%); Country- or Study-specific percentiles (12, 7.5%); BMI, WH, WL ≥97% for age and gender (11, 6.9%); International Obesity Task Force Percentiles (10, 6.3%); Cole et al. BMI percentiles (8, 5.0%); BMIZ, WLZ, WHZ ≥+3 (5, 3.1%); BMI ≥30 (1, 0.6%) Unknown (8, 5.0%)
**Elevated Waist Circumference (n = 43)**	WC ≥90% for age, height and gender (29, 67.4%); WC ≥75% for age, height and gender (5, 11.6%); Country- or Study-Specific Percentiles (4, 9.3%); WC ≥80% for height and age (2, 4.6%); Cutoffs proposed by Taylor et al. (2, 4.6%); WC ≥80 and 90 cm in females and males respectively (2, 4.6%); Cutoffs defined from World Health Organization 1988 (1, 2.3%); Ratio of WC:height ≥0.55 (1, 2.3%)
**Glucose Intolerance (n = 27)**	Fasting glucose ≥100 mg/dL (21, 77.8%); Fasting glucose ≥110 mg/dL (4, 14.8%); Fasting glucose ≥126 mg/dL (1, 3.7%); Fasting glucose ≥5.55 mmol/L (1, 3.7%)
**Elevated Triglycerides (n = 30)**	TG ≥150 mg/dL (10, 33.3%); TG ≥100 mg/dL in children age 2–9 yo (10, 33.3%); TG ≥ 130 mg/dL in children ≥10 yo (7, 23.3%); TG ≥110 mg/dL (6, 20.0%); TG ≥1.96 mmol/L (1, 3.3%); TG ≥1.5 mmol/L (1, 3.3%); TG ≥75 mg/dL (1, 3.3%); TG ≥95% for age and gender (1, 3.3%); TG ≥90% for age and gender (1, 3.3%); TG ≥75≤99 mg/dL for ages 0–9 and ≥90≤125 mg/dL for ages 15–19 (1, 3.3%); TG >100 mg/dL for age 2–15 and ≥125 for age 15–19 (1, 3.3%); unknown (1, 3.3%)
**Low HDL Cholesterol (n = 38)**	HDL ≤40 mg/dL (18, 47.4%); HDL ≤45 mg/dL (9, 23.7%); HDL ≤35 mg/dL (4, 10.5%); HDL ≤50 mg/dL (1, 2.6%); HDL ≤50 mg/dL for females and HDL ≤40 mg/dL for males (1, 2.6%); HDL ≤1.2 mmol/L (1, 2.6%); HDL ≤1.09 mmol/L (1, 2.6%); HDL ≤ 0.9 mmol/L (1, 2.6%); HDL <10% for age and gender (1, 2.6%); HDL ≤38 mg/dL for females and ≤36 mg/dL for males (1, 2.6%)
**High Blood Pressure (n = 33)**	SBP or DBP ≤90% for age, height and gender (22, 66.7%); SBP ≥130 mmHg (4, 12.1%); DBP ≥85 mmHg (4, 12.1%); SBP or DBP ≥95% for age, height and gender (4, 12.1%); American Pediatric Society guidelines (1, 3.0%); Argentinean national guidelines (1, 3.0%)

BMI = body mass index; WH = waist to height ratio; WL = weight to length ratio; WC = waist circumference; TG = triglycerides; HDL = high density lipoprotein; SBP = systolic blood pressure; DBP = diastolic blood pressure.

### Obesity

**Fig 2:**
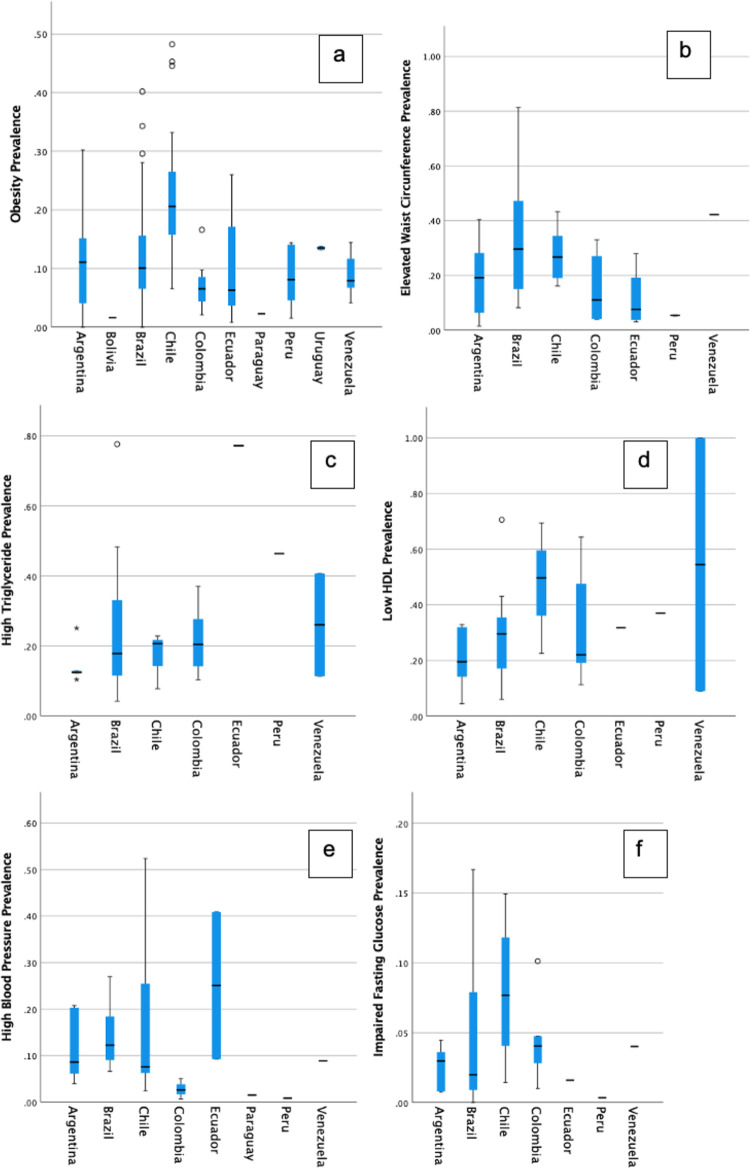
Cardiometabolic risk factor prevalence in South American children by country. Box-and-whisker plots showing median and IQR values for each CMRF by country by any definition. Y-axis is prevalence, x-axis is country. A) obesity, B) elevated waist circumference, C) high triglyceride, D) low HDL, E) high blood pressure, F) impaired fasting glucose. HDL = high-density lipoprotein.

Results are summarized in Table 4. One hundred and fifty-nine studies (94.4% of included studies) measured obesity; these included 2,787,579 individuals and 15 different definitions. The most commonly used definitions of obesity were BMI, WH (waist-to-height ratio), or WL (waist-to-length ratio) ≥95% for age and gender (53 studies, 33.3%). In total by any definition, a median of 12.2% (Interquartile range (IQR) 6.35–17.2) children were obese with 23.9% (IQR 16.6–39.0) with overweight and/or obesity without distinction, and a median of 21.9% (IQR 10.0–33.2) specifically experiencing abdominal obesity. Eight studies (5.0%) included indigenous populations; obesity by any definition was lower in indigenous children than non-indigenous children (median, IQR: 2.5%, 1.6–5.6 vs. 12.7%, 6.5–17.5, p = 0.009). Obesity by any definition varied significantly by country, with Chile exhibiting the highest prevalence of both obesity and obesity and/or overweight without distinction (median, IQR obesity in Chile: 20.6%, 15.6–27.8 and obesity/overweight: 47.5%, 41.1–60.8) when compared to other countries (Table 4, Fig 2). Obesity by any definition was lower in rural populations than in urban or mixed populations (median, IQR obesity in rural setting: 5.6%, 1.6–12.0; in urban setting: 12.6%, 6.3–17.3; and mixed setting: 12.9%, 6.5–16.2; p = 0.08).

**Table 4:** Cardiometabolic risk factors in South American children by any definition and by the most common definition, study setting, country, and inclusion of indigenous population.

		Obesity by any definition			Obesity by BMI, WH, WL ≥95%*		
Variables		N (%)	Prevalence %, median (IQR)	p value	N (%)	Prevalence %, median (IQR)	p value
**Overall**		2787579	12.2 (6.3–17.3)		217989	13.4 (7.0–20.6)	
**Setting**	Urban	105643	12.6 (6.5–18.0)	0.08	63398	13.9 (6.8–20.4)	0.08
	Mixed	2672381	12.9 (6.5–16.2)		147401	13.6 (7.2–17.6)	
	Rural	8702	5.6 (1.6–12.0)		7190	4.9 (2.0–9.1)	
**Country**	Argentina	1400	11.1 (3.4–16.3)	<0.001	6567	11.1 (4.4–14.6)	0.03
	Bolivia	556	1.6				
	Brazil	179591	10.1 (6.5–15.9)		17609	12.8 (7.7–18.0)	
	Chile	192333	20.6 (15.6–27.8)		183393	21.4 (13.7–28.5)	
	Colombia	12633	6.5 (4.1–9.7)		2628	7.4 (2.1)	
	Ecuador	13523	6.3 (2.6–17.3)		7292	5.8 (3.6)	
	Paraguay	132	2.3				
	Peru	2368236	8.1 (4.2–14.1)		500	14.4	
	Uruguay	4048	13.2 (11.6)				
	Venezuela	1674	7.3 (4.8–12.8)				
**Indigenous Population**	Yes	1554	2.5 (1.6–5.6)	0.009	812	3.4 (1.6)	0.007
	No	2786025	12.7 (6.5–17.5)		217177	13.9 (7.7–21.1)	
** **		**Obese and/or overweight by any definition**			**Obesity and/or overweight defined as BMI,WH,WL ≥95%**		
**Variables**		**N (%)**	**Prevalence %, median (IQR)**	**p value**	**N (%)**	**Prevalence %, median (IQR)**	**p value**
**Overall**		504853	23.9 (16.5–39.3)		502726	22.9 (18.0–35.6)	
**Setting**	Urban	170723	26.3 (18.3–41.3)	0.01	116546	26.3 (19.0–35.1)	0.003
	Mixed	324007	22.8 (16.6–35.7)		130558	20.4 (12.0–24.1)	
	Rural	10123	16.2 (10.8–23.6)		8518	10.9 (6.7–16.1)	
**Country**	Argentina	13739	21.1 (10.1–32.6)	<0.001	6135	18.4 (10.9–29.8)	0.008
	Bolivia	2873	10.6 (10.5)				
	Brazil	201223	24.0 (17.7–36.2)		28563	22.8 (16.8–34.6)	
	Chile	166303	47.5 (41.1–60.8)		157609	50.2 (35.0–64.2)	
	Colombia	73249	18.7 (11.9–23.4)		55326	18.1 (13.4–22.6)	
	Ecuador	13720	19.9 (13.5–26.4)		7489	24.7 (21.0–81.6)	
	Paraguay	132	10.6				
	Peru	31447	21.7 (20.5–31.6)		500	22	
	Uruguay	103	27.2				
	Venezuela	2064	20.8 (15.2–29.0)				
**Indigenous Population**	Yes	2975	10.8 (7.2–12.9)	<0.001	2140	10.9 (6.6–12.2)	<0.01
	No	501878	25.2 (17.9–40.1)		253482	24.7 (19.9–38.2)	
** **		**Elevated Waist Circumference by any definition**			**Elevated Waist Circumference defined as WC ≥90% for age and gender**		
**Variables**		**N (%)**	**Prevalence %, median (IQR)**	**p value**	**N (%)**	**Prevalence %, median (IQR)**	**p value**
**Overall**		38250	21.9 (9.4–33.9)		22865	10.6 (5.4–27.7)	
**Setting**	Urban	25416	24.0 (8.6–38.1)	0.55	17934	10.6 (5.2–28.2)	0.40
	Mixed	10166	30.4 (10.9–41.0)		4099	5.6 (3.0)	
	Rural	2668	16.3 (10.7–23.8)		832	16.3 (6.4)	
**Country**	Argentina	4211	19.2 (5.1–31.2)	0.04	4211	19.2 (5.1–31.3)	0.26
	Brazil	14410	29.6 (10.5–48.0)		4773	20.1 (10.2–43.6)	
	Chile	6757	26.7 (18.7–35.1)		1686	26.0 (16.2)	
	Colombia	10337	11.1 (4.1–28.5)		9750	5.6 (4.1–22.0)	
	Ecuador	1359	7.6 (3.4–23.6)		1359	7.6 (3.4–23.6)	
	Peru	1086	5.4 (5.2)		1086	5.4 (5.2)	
	Venezuela	90	42.2				
**Indigenous Population**	Yes	423	10.7 (6.4)	0.28			
	No	37827	24.0 (10.0–35.1)				
** **		**Impaired Fasting Glucose by any definition**			**Impaired Fasting Glucose by Glucose ≥100 mg/dL**		
**Variables**		**N (%)**	**Prevalence %, median (IQR)**	**p value**	**N (%)**	**Prevalence %, median (IQR)**	**p value**
**Overall**		19810	3.0 (1.0–6.6)		15263	2.8 (1.0–6.7)	
**Setting**	Urban	11941	3.2 (1.5–6.2)	0.92	10908	2.8 (1.4–6.7)	0.10
	Mixed	5076	3.7 (0.6–8.8)		2064	4.8 (0.9)	
	Rural	2793	3.0 (1.0)		2291	2.0 (1.0)	
**Country**	Argentina	4230	3.0 (0.8–4.0)	0.44	2017	1.9 (0.8)	0.44
	Brazil	4523	2.0 (0.9–8.6)		3030	1.4 (0.6–5.8)	
	Chile	2919	7.7 (2.7–13.4)		2919	7.7 (2.7–13.4)	
	Colombia	6000	4.0 (1.9–7.4)		6000	4.0 (1.9–7.4)	
	Peru	586	0.3		375	1.6	
	Venezuela	1012	4		922	4	
		**Low HDL by any definition**			**Low HDL by HDL ≤40 mg/dL**		
**Variables**		**N (%)**	**Prevalence %, median (IQR)**	**p value**	**N (%)**	**Prevalence %, median (IQR)**	**p value**
**Overall**		85802	29.6 (17.7–36.9)		15181	22.6 (19.2–36.7)	
**Setting**	Urban	13482	28.8 (19.5–36.4)	0.27	8259	22.1 (19.3–36.1)	0.14
	Mixed	67044	37.0 (14.1–69.5)		1983	40.1 (37.0)	
	Rural	5276	16.4 (7.9–32.5)		4939	24.2 (12.6–32.7)	
**Country**	Argentina	5184	19.6 (11.7–32.3)	0.62	3866	25.9 (19.5–32.7)	0.82
	Brazil	71797	29.6 (17.0–35.8)		5015	35.5 (13.2–39.9)	
	Chile	1210	49.7 (22.6)		208	22.6	
	Colombia	6000	22.1 (15.3–56.0)		5506	20.6 (13.3–53.8)	
	Peru	586	37		586	37	
	Venezuela	1003	59.7 (9.0)				
		**Elevated Triglycerides by any definition**			**Elevated Triglycerides defined as ≥100 mg/dL for ages 2–9 and ≥130 mg/dL for ages 10–19**		
**Variables**		**N (%)**	**Prevalence %, median (IQR)**	**p value**	**N (%)**	**Prevalence %, median (IQR)**	**p value**
**Overall**		84384	18.1 (11.6–34.9)		68166	19.1 (10.4–26.2)	
**Setting**	Urban	13336	19.4 (12.7–31.3)	0.76	1611	20.4 (11.0–25.1)	0.62
	Mixed	66677	12.3 (8.3–54.2)		63927	44.9 (12.2)	
	Rural	4371	17.7 (10.3–42.5)		2628	10.4 (10.4)	
**Country**	Argentina	4290	12.4 (11.3–19.0)	0.51	1346	17.7 (10.4)	0.85
	Brazil	71273	17.8 (10.9–36.7)		64836	17.8 (8.2–53.6)	
	Chile	1210	20.7 (7.8)		208	15.4 (10.4)	
	Colombia	6000	20.4 (12.3–32.4)		1282	10.4	
	Peru	586	46.4				
	Venezuela	1003	26.1 (11.3)				
		**High blood Pressure by any definition**			**High Blood Pressure by BP ≥90% for age, gender, and height**		
**Variables**		**N (%)**	**Prevalence %, median (IQR)**	**p value**	**N (%)**	**Prevalence %, median (IQR)**	**p value**
**Overall**		21055	8.6 (4.6–20.6)		12784	8.6 (4.2–20.0)	
**Setting**	Urban	6957	8.7 (6.0–22.0)	0.73	5818	8.7 (6.0–29.3)	0.67
	Mixed	10938	9.3 (3.6–22.1)		5313	7.6 (2.0–36.9)	
	Rural	2372	8.0 (2.5–15.0)		1653	8.0 (4.2)	
**Country**	Argentina	7864	8.6 (4.2–20.5)	0.09	4215	8.0 (4.1–14.3)	0.19
	Brazil	5991	14.3 (8.0–25.4)		3461	15.9 (11.3)	
	Chile	3076	7.6 (5.3–32.2)		2286	7.8 (4.3–38.9)	
	Colombia	2270	2.6 (0.7)		988	3.8 (2.6)	
	Ecuador	335	25.1 (9.3)		335	25.1 (9.3)	
	Paraguay	132	1.5				
	Peru	586	0.8		586	0.8	
	Venezuela	913	0.9		913	0.9	

IQR = inter-quartile range; p-values calculated to 95% significance; BMI = body mass index; WH = waist to height ratio; WL = weight to length ratio; HDL = high density lipoprotein.

When looking only at obesity as defined by BMI, WH or WL ≥95%, this was higher in non-indigenous populations than indigenous populations (median, IQR for non-indigenous: 13.9%, 7.7–21.1 vs. for indigenous: 3.4%, 1.6, n = 2; p = 0.07) and differed by setting and country (Table 4). Rural populations experienced lower rates of obesity by this definition than did mixed and urban populations, which were almost equivalent (median, IQR: rural 4.9%, 2.0–9.1 vs. mixed 13.6%, 7.2–17.6 vs. urban 13.9%, 6.8–20.4, p = 0.08). These differences held for those studies in which obesity was indistinguishable from overweight (Table 4).

### Elevated waist circumference

Elevated waist circumference was reported in 43 studies (24.0% of total studies included), encompassing 38,250 individuals. The most commonly used definition of waist circumference was WC ≥90% for age, height and gender (29 studies, 67.4%). Brazil had the highest prevalence of elevated waist circumference by any definition (median, IQR: 29.6%, 10.5–48.0, p = 0.04). There were no differences in median prevalence rates of elevated waist circumference defined by WC ≥90% for age and gender between setting nor by country (Argentina, Brazil, Chile, Colombia, and Ecuador; Table 4). There was a trend to higher median prevalence in Chile and a lower prevalence in Colombia and Peru (median, IQR for Chile: 26.0%, 16.2 n = 2 vs. Colombia: 5.6%, 4.1–22.0 vs. Peru: 5.4%, 5.2% n = 2) as detailed in Fig 2.

### Impaired fasting glucose

Twenty-seven studies (15.1% of included studies) measured impaired fasting glucose and included 19,810 individuals using 4 different definitions (Table 3). The most common definition was fasting glucose ≥100 mg/dL (21, 77.8%). The median prevalence of impaired fasting glucose by any definition was 3.0% (IQR 1.0–6.6), which was comparable to the median prevalence of impaired fasting glucose by the most commonly used definition (2.8% IQR 1.0–6.7). For both impaired fasting glucose overall and studies that used the most common definition, studies with mixed settings experienced slightly higher prevalence rates over studies with strictly urban and rural settings (Table 4). Chile trended to a higher median prevalence of impaired fasting glucose than other countries in the analysis, but this did not reach statistical significance (Table 4).

We also recorded the prevalence of insulin resistance and hyperinsulinemia in studies that reported these risk factors. The median prevalence rates of hyperinsulinemia and insulin resistance by any definition was 6.3% (IQR 3.3–12.7) and 5.4% (IQR 4.8–27.3), respectively. The number of studies that reported on these risk factors (n = 7, 3.9% of included studies) for hyperinsulinemia, n = 9 (5.0%) for insulin resistance) was too small to complete summary statistics on this data.

### High blood pressure

Thirty-three studies measured high blood pressure (18.5% of included studies) encompassing 21,055 individuals and 8 different definitions (Table 3). The most common definition of HBP was SBP or DBP ≥90% for age, height and gender (22, 66.7%). The median prevalence of high blood pressure by any definition was 8.6% (IQR 5.1–20.6), and the median prevalence as defined by BP ≥90% for age, gender and height was 8.6% (IQR 4.2–20.0). HBP did not differ greatly between study setting or country, although Ecuador exhibited a higher trend in HBP than other countries (median, IQR 25.1%, 9.3 n = 2). Prevalence rates are detailed in Table 4.

### Dyslipidemia

Thirty-eight studies (21.2% of studies included) measured low HDL including 85,802 individuals from 6 countries using 9 different definitions (Table 3). Thirty studies (16.8% of included studies) measured elevated triglycerides including 84,384 individuals from 6 countries using 12 different definitions. Data is summarized in Table 4.

#### Elevated triglycerides

Less than 20% of South American children experienced elevated triglycerides (TG) (median, IQR 18.1%, 11.6–34.9) by any definition; this was similar to the prevalence as defined by the most common definitions of TG ≥100 mg/dL in children age 2–9 years together with TG ≥130 mg/dL in children 10–19 years (median, IQR 19.1%, 10.4–26.2). There were no notable differences in hypertriglyceridemia between children in different study settings, though children in mixed settings in studies that used the two most common definitions had slightly higher levels of TG than children in strictly urban and rural settings (median, IQR in mixed: 44.9%, 12.2 n = 2; vs. urban: 20.4%, 11.0–25.1 vs. rural: 10.4%, n = 1). Venezuelan children had slightly higher prevalence of elevated TG than children in other countries, but this did not reach statistical significance (median, IQR 26.1%, 11.3 n = 2, Table 4).

#### Low HDL cholesterol

The most commonly used definition of low HDL was HDL≤40 mg/dL (18 studies, 47.4% of studies reporting on dyslipidemia; Table 3). The median prevalence of low HDL cholesterol by any definition was 29.6% (IQR 17.7–36.9) and 22.6% (IQR 19.2–36.7) as defined by HDL ≤40 mg/dL. Low HDL did not vary greatly by either study setting or country for all studies or for those that used the most common definition (HDL ≤40 mg/dL) (see Table 4). Rural populations experienced a slightly lower prevalence of low HDL in all studies (median, IQR 16.4%, 7.9–32.5) versus mixed and urban populations (median, IQR mixed: 37.0%, 14.4–69.5 vs. urban: 28.8%, 19.5–36).

## Discussion

### Comparison to other literature

Our study provides the most comprehensive assessment of CMRF in South American children. Krishnan and Short [191] assessed CMRF prevalence in South American children with type 1 diabetes mellitus, but we did not find any other articles that focused on the general South American child and adolescent population. In comparison to other geographic populations, CMRF prevalence in South American children, and children in general, is poorly characterized. We could find few meta-analyses that focus on regional burdens of CMRF prevalence; many focus on a subset of the child and adolescent population, such as Krishnan and Short, or are concerned with assessing screening tools [191]. Most regionally-focused literature on CMRF in children and adolescents details obesity and overweight prevalence, while dyslipidemia and impaired glucose metabolism are minimally characterized. The World Health Organization estimates that as of 2016, roughly 12.8% of children and adolescents aged 10–19 in the Americas experience obesity, the highest of all regions [192]. The next-closest region is the Western Pacific, with 7.4% of children experiencing obesity, which demonstrates how alarmingly high the prevalence of childhood and adolescent obesity is in the Americas as compared to the rest of the globe and the need to focus additional attention in this vulnerable region. In addition, we chose to focus the scope of this article on South America. In our preliminary investigation of this topic, we found a paucity of information on CMRF in Caribbean children.

### Controversy of metabolic syndrome in children

Although the utility and consistency of a diagnosis of MetS in children is controversial, a more comprehensive screening tool for population-level prevalence of cardiometabolic health factors does not yet exist. Many organizations and publications, including the AHA, have questioned the utility of diagnosing MetS in children. Goodman et al, 2007 showed that potentially large proportions of children diagnosed with MetS do not meet criteria upon follow-up 3 to 6 years later [193]. On the other hand, there is literature to support an increased risk of persistence of MetS into adulthood [194]. In one cohort study of 771 adults (mean age 38 years) who had previously participated in the Lipid Research Clinics study as children and adolescents, the incidence of self-reported cardiovascular disease was more common in adults who exhibited metabolic syndrome traits as children than in those who did not (19.4 versus 1.5%, odds ratio 14.6, 95% CI 4.8–45.3) [195]. Of 31 children who had metabolic syndrome traits as children, 21 (68%) had metabolic syndrome as adults. In the context of these results, the high burden of obesity and dyslipidemia demonstrated in our analysis suggests a future rise in CMRF in the South American adult population.

### Limitations

The greatest limitation in interpreting this data is the heterogeneity of definitions used for individual CMRF. As displayed in Table 2, there were 4–15 definitions used for each risk factor. We addressed this issue by generating additional summary statistics with data that shared the same cutoff definition. This issue would be avoided in future studies if there was an international consensus on and wider acceptance of standardized definitions for CMRF in children.

We could not find any published data from Guyana, Suriname, French Guiana nor the Falkland Islands. The absence of data from these countries and territories limits the generalizability of this study to the whole region. Additionally, we extracted as much demographic information as possible; however, we were unable to extract basic demographic data such as gender for each definition, as many studies reported more than one risk factor but not necessarily gender distribution or ages per risk factor. Therefore, we do not know if CMRF are clustered more in one gender over another or one age group over another. A further limitation was the minimal inclusion of indigenous or other minority populations. Although many South American populations include indigenous members, only a handful of studies specifically noted the inclusion of majority indigenous groups and no studies specifically reported on other minority groups. Our analysis therefore likely underestimates the prevalence of CMRF in these more vulnerable individuals.

Additionally, when compiling the titles from the basic Google Scholar search, there was a server error after uploading 980 titles from this database despite multiple attempts to upload all results. Therefore, only 980 of the 1,060 titles from this search were able to be included for screening. It is unknown how many of the eighty titles would have met our inclusion criteria. Titles from Google Scholar were imported using the Mendeley plug-in for Google Chrome. This software did not differentiate between Spanish and English versions of the same text, so there were many duplicate titles that were imported and had to be screened out. This led to a high number of duplicate titles.

### Collective trends

Of all CMRF, obesity was the most widely described and thus generated the largest differences between variables (ex. study setting and indigenous populations). This is likely due to sample size, and it is possible that the variables evaluated for other CMRF are underpowered. Low HDL and overweight and/or obese status were the two most prevalent CMRF, and impaired fasting glucose and HBP were the lowest. These trends are consistent with the results of Li et al., who found similar rates of CMRF in severely obese children in the United States (HBP: 9.9%, low HDL: 40%, high TG: 30.0%) [196]. The higher rates of obesity and overweight are not surprising, though the high prevalence of HDL was not expected. This finding could be influenced by the growing urbanization of South America resulting in increased consumption of processed foods and sedentary lifestyle of South American children in mixed and urban areas [197].

Brazil, Chile, Colombia, and Argentina were the most represented in terms of number of studies. Though Peru had the largest population included in our analysis, this is due to one study (Torres-Roman et al.) including a population of over 2 million. Our study highlights an especially high prevalence of many CMRF in Chilean children.

Overall, children in rural settings and indigenous children trended to lower prevalence rates of CMRF. This differs from other studies on the nutritional status of indigenous populations worldwide, which document a growing double burden of undernutrition and obesity in indigenous children [198]. This inconsistency could be attributed to the small sample sizes and lack of available data on indigenous populations in South America.

### Knowledge gaps

Many South American countries were underrepresented or not represented at all in our analysis due to a lack of available data. There were three or fewer studies that contained data from Bolivia, Paraguay, and Uruguay for each CMRF, and many countries only had data reported on obesity. Many countries, including French Guiana and Suriname, did not have any available or relevant articles. As outlined above, indigenous and minority populations should be the focus of future studies, as they were vastly underrepresented in our comprehensive analysis.

## Conclusion

The high prevalence of obesity, overweight, and dyslipidemia in South American children are concerning. This study provides strong evidence to policy makers and healthcare workers to design interventions to address CMRF clustering in children to curb future disease burden. Our study also denotes the analytical difficulties that arise from a lack of standardized and widely accepted CMRF definitions in children. Additionally, future studies should focus on characterizing CMRF in underrepresented countries and populations, delineating the burden distribution by gender and age, and confirming the finding here of higher clustering of CMRF in urban populations. It is our hope that this meta-analysis will stimulate additional research to stem the rapid regional and global rise in childhood metabolic syndrome and early cardiovascular disease.

## Supporting information

S1 Checklist PRISMA 2020 checklist. (DOCX)

## Abbreviations

CMRF: Cardiometabolic risk factors
HBP: High blood pressure
MetS: Metabolic syndrome
IDF: International Diabetes Foundation
AHA: American Heart Association
BMI: Body mass index
WC: Waist circumference
GI: Glucose Intolerance
HDL: high-density lipoprotein
LDL: Low-density lipoprotein
WHO: World Health Organization
