# Supplementary material for: Cardiometabolic risk factors in South American children: A systematic review and meta-analysis
Source: PLoS One. 2023 Nov 22;18(11):e0293865. doi: 10.1371/journal.pone.0293865 (PMC10664905; doi:10.1371/journal.pone.0293865)
Supplement: S1 Checklist — (DOCX) [file pone.0293865.s001.docx]

| **Section and Topic** | **Item #** | **Checklist item** | **Location where item is reported** |
| --- | --- | --- | --- |
| **TITLE** | | |  |
| Title | 1 | Identify the report as a systematic review.  **In a review quantifying the cardiometabolic risk burden in South American children, the authors identify the report as a systematic review:**  “Cardiometabolic risk factors in South American children: a systematic-review and meta-analysis” | Page 1, title |
| **ABSTRACT** | | |  |
| Abstract | 2 | See the PRISMA 2020 for Abstracts checklist.  **In a review examining cardiometabolic risk factor prevalence in South American children, the authors summarized the objectives, eligibility criteria, databases consulted, methods for collecting data, assessing risk of bias and synthesising results, along with presenting results and commenting on the limitations of the evidence.**  “**Background:** Cardiometabolic risk factors (impaired fasting glucose, abdominal obesity, high blood pressure, dyslipidemia) cluster in children, may predict adult disease burden, and are inadequately characterized in South American children. **Objectives:** To quantify the burden of cardiometabolic risk factors in South American children (0-21 years) and identify knowledge gaps **Methods:** We systematically searched PubMed, Google Scholar, and the Latin American and Caribbean Health Sciences Literature via Virtual Health Library from 2000-2021 in any language. Two independent reviewers screened and extracted all data. **Results:** 179 studies of 1,540 screened were included representing 10 countries (n=2,975,261). 12.2% of South American children experienced obesity, 21.9% elevated waist circumference, 3.0% elevated fasting glucose, 18.1% high triglycerides, 29.6% low HDL cholesterol, and 8.6% high blood pressure. Cardiometabolic risk factor definitions varied widely. Chile exhibited the highest prevalence of obesity/overweight, low HDL, and impaired fasting glucose. Ecuador exhibited the highest prevalence of elevated blood pressure. Rural setting (vs. urban or mixed) and indigenous origin protected against most cardiometabolic risk factors. **Conclusions:** South American children experience high rates of obesity, overweight and dyslipidemia. International consensus on cardiometabolic risk factor definitions for children will lead to improved diagnosis of cardiometabolic risk factors in this population, and future research should ensure inclusion of unreported countries and increased representation of indigenous populations.” | Page 2, “ABSTRACT” Lines 50-67 |
| **INTRODUCTION** | | |  |
| Rationale | 3 | Describe the rationale for the review in the context of existing knowledge.  **In a review examining cardiometabolic risk factor prevalence in South American children, the authors acknowledge the knowledge gap and limitations of current literature:**  “Metabolic syndrome is the clustering of cardiometabolic risk factors (CMRF) of impaired fasting glucose, abdominal obesity, high blood pressure (HBP), and dyslipidemia.(1) As of 2017, 20-25% of the world’s population was estimated to have metabolic syndrome (MetS) with an associated two or three times increased risk of death by heart attack or stroke respectively.(1) Childhood obesity is on the rise globally with an inconsistent understanding of the burden and impact of MetS in childhood and beyond.(2) Children with obesity are more likely to have obesity in adulthood, which is itself associated with significant health complications including gallstones, type 2 diabetes mellitus, non-alcoholic fatty liver disease, osteoarthritis, certain cancers, and cardiovascular events.(3) A rapid rise in obesity over the past 50 years has led to an almost 40% or 70% rise in costs of healthcare provision and treatment, respectively, and this now outweighs spending on non-communicable diseases associated with tobacco use or alcohol dependence.(3)  **Defining Cardiometabolic Risk Factors and Metabolic Syndrome in Children**  More than 40 different definitions of MetS in the pediatric population exist.(4) Two notable attempts to approach MetS definition in the pediatric population have been made by the International Diabetes Foundation (IDF) and the American Heart Association (AHA).(4) The IDF definition states that the adult definition can be used down to the age of 10, with the only modification being a waist circumference above the 90th percentile as the abdominal obesity parameter rather than using adult cutoffs. The AHA did not explicitly create their own guidelines, but rather pointed to the conclusions of three studies, Cook et al, de Ferranti et al, and Ford et al, as appropriate starting points.(4–7)  Many organizations and publications, including the AHA, have questioned the utility of diagnosing MetS in children. Goodman et al, 2007 showed that potentially large proportions of children diagnosed with MetS do not meet criteria upon follow up 3 to 6 years later.(8) Although the utility and stability of an individual’s pediatric diagnosis of MetS is questionable, there has yet to be a more comprehensive screening tool for population-level prevalence of cardiometabolic health factors, which is why the authors chose to evaluate the CMRF for this study in the context of MetS. Viewing CMRFs in this way allows for risk factors to be seen as clusters, which can give insight into targeted population-specific approaches to potentially reduce future disease burden.  MetS is highly unstable throughout childhood; a child can meet the criteria at one point in time but not later, and it is unclear whether this variation represents an improvement or deterioration in health status.(9) In one cohort study of 771 adults (mean age 38 years) who had participated in the Lipid Research Clinics study as children and adolescents previously, the incidence of self-reported cardiovascular disease was more common in adults who exhibited metabolic syndrome traits as children than in those who did not (19.4 versus 1.5%, odds ratio 14.6, 95% CI 4.8-45.3).(10) Of 31 children who had metabolic syndrome traits as children, 21 (68%) had metabolic syndrome as adults. Increasing body mass index (BMI) was strongly associated with a risk of adult metabolic syndrome.  The rise in global obesity and CMRF is particularly relevant in Latin American and Caribbean countries, where cardiovascular disease is now the most common cause of death and disability.(11) Despite the increasing attention on CMRF globally and their short and long term effects both in populations and in an individual’s lifespan, the burden of CMRF in South American children is poorly defined and scholarly articles about prevalence of CMRF in this population are scarce.(12) A recent search on PubMed using the terms “(cardiometabolic risk OR metabolic syndrome) AND children Latin America” only yielded 16 results, less than half of which were relevant to the topic. Filling this knowledge gap would likely contribute to reducing this disease burden by illuminating the most common CMRF and bringing policy and healthcare attention to diagnosis, prevention, and treatment of at-risk children.” | Page 3-5, “INTRODUCTION” Lines 70-118 |
| Objectives | 4 | Provide an explicit statement of the objective(s) or question(s) the review addresses.  **In a review quantifying the cardiometabolic risk burden in South American children, the authors report the objectives of the review as the following:**  “The objective of this systematic review and meta-analysis was to quantify the burden of cardiometabolic risk factors in South American children, identify knowledge gaps, and propose next steps for research.” | Page 5, “Objectives” Lines 119-121 |
| **METHODS** | | |  |
| Eligibility criteria | 5 | Specify the inclusion and exclusion criteria for the review and how studies were grouped for the syntheses.  **In a review examining the cardiometabolic risk factor prevalence in South American children, the authors report the types of studies, participants, and outcomes that were eligible for inclusion in the review, indicating that studies were excluded if they were published before the year 2000 and if the study population was skewed to over-represent known cardiometabolic risk factors (obese individuals, children with diagnosed dyslipidemia, etc.).**  “**Inclusion Criteria:** We included all primary quantitative data from human subject studies on children ages 0-21 years from the geographical region of South America (excluding Caribbean islands) reported in any language. We included studies that reported prevalence data using any definition on at least one of the following: glucose intolerance; obesity; elevated waist circumference (WC); high blood pressure; and/or dyslipidemia, considered as low high-density lipoprotein (HDL), high low-density lipoprotein (LDL), and/or high triglyceride (TG). **Exclusion Criteria:** We excluded studies without available data, with data collected before 2000, focusing on a population with a chronic and/or congenital medical condition, duplicate data across multiple publications, duplicate data reported in different languages, and with cutoff definitions for CMRF that could not be obtained. Studies who recruited participants solely based on overweight/obese status were excluded in order to minimize selection bias, as CMRF are higher in obese populations.” | Page 5  “METHODS” Lines 125-136 |
| Information sources | 6 | Specify all databases, registers, websites, organisations, reference lists and other sources searched or consulted to identify studies. Specify the date when each source was last searched or consulted.  **In a review exploring the cardiometabolic risk burden in South American children, the authors list the electronic bibliographic databases and websites searched. More information is available in a table.**  “An electronic search was performed using the databases PubMed, the Latin American and Caribbean Health Sciences Literature via Virtual Health Library, and Google Scholar. We examined the grey literature through the Google Scholar search.” | Page 6  “Search Strategy”  Lines 145-147 |
| Search strategy | 7 | Present the full search strategies for all databases, registers and websites, including any filters and limits used.  **In a review quantifying the cardiometabolic risk factor prevalence in South American children, the authors report the full search strategy for PubMed, the Latin American and Caribbean Health Sciences Literature via Virtual Health Library, and Google Scholar, along with the list of terms used when searching the websites. More information is available in a table.**  “Our search strategy is detailed in Table 1. We searched all available medical literature, including gray literature, using keyword searches in relevant databases. Our search strategy was composed of three main steps: 1) we performed an initial search of primary literature aggregators, ex. PubMed, to determine appropriate keyword terms in titles and abstracts; 2) we then used appropriate keywords to conduct a more thorough search of the literature; finally, 3) we examined references of articles found in step two to identify additional relevant data sources. We used Preferred Reporting Items for Systematic Review and Meta-analyses Protocols (PRISMA-P) as a framework to develop and guide our review.(13) An electronic search was performed using the databases PubMed, the Latin American and Caribbean Health Sciences Literature via Virtual Health Library, and Google Scholar. We examined the grey literature through the Google Scholar search. The initial search terms included: “*Cardiometabolic AND risk factor AND Latin America NOT adult,” “Cardiometabolic risk factor Ecuador,”* and *“Metabolic syndrome [MeSH Term] AND Latin America [MeSH Term].”* MeSH terms were used when searching PubMed. The keywords around which the searches were developed included phrases revolving around CMRF, children, adolescents and South America or Ecuador. This search strategy, specifically in Google Scholar, did not adequately represent all South American countries. Therefore, we expanded the Google Scholar search with advanced search features to combine all terms for each CMRF and individually searching all spelling variations for each South American country plus “children”. Figure 1 shows the PRISMA flow chart and screening process of all articles that the first search strategy yielded.” | Page 6  “Search Strategy”  Lines 138-157 |
| Selection process | 8 | Specify the methods used to decide whether a study met the inclusion criteria of the review, including how many reviewers screened each record and each report retrieved, whether they worked independently, and if applicable, details of automation tools used in the process.  **In a review examining the cardiometabolic risk factor burden in children in South America, the authors report piloting, double screening and consensus methods for study selection:**  “One reviewer (CH) screened titles and abstracts of the retrieved articles for relevance, and a second reviewer (SB) independently confirmed relevance. Disputes were settled by an arbitrator (SLA). Figure 1 shows the associated PRISMA flowchart of study selection. Two independent people (CH, SB, LD, and/or NR) performed data extraction and assessment of study quality.” | Page 9  “Study Selection”  Lines 168-172 |
| Data collection process | 9 | Specify the methods used to collect data from reports, including how many reviewers collected data from each report, whether they worked independently, any processes for obtaining or confirming data from study investigators, and if applicable, details of automation tools used in the process.  **In a review exploring cardiometabolic risk factor prevalence in South American children, the authors report using a data collection form, the number of authors collecting data from studies and the process of resolving disagreements, and indicate that the study authors were contacted if any data were unclear.**  “After comparison of extracted data, disputes were settled through an arbitrator (SLA). For studies that included an intervention, only baseline data was recorded, so that interventions could not skew initial population prevalence of CMRF. Study authors were contacted if any data was unclear.” | Page 10  “Study Selection”  Lines 172-175 |
| Data items | 10a | List and define all outcomes for which data were sought. Specify whether all results that were compatible with each outcome domain in each study were sought (eg. for all measures, time points, analyses), and if not, the methods used to decide which results to collect.  **In a review quantifying the burden of cardiometabolic risk factors in children in South America, the authors list and define the outcomes for which data were sought (ex. Obesity measurements, dyslipidemia measurements, blood pressure) and specify the decision rules used to decide which results to collect when multiple were available in studies (ex. When multiple measures, time points and unadjusted and adjusted analyses were available).**  “Primary outcomes were prevalence of CMRF and study quality. For studies that reported CMRF prevalence at multiple time points, the most recent timepoint was recorded. CMRF definitions used by each study and their corresponding data were also recorded. Secondary outcomes were demographic data and study characteristics including age, rural vs. mixed vs. urban study setting, inclusion of indigenous population, year(s) of data collection, gender distribution, study location, study design, and study inclusion and exclusion criteria.” | Page 10  “Outcomes”  Lines 177-182 |
|  | 10b | List and define all other variables for which data were sought (e.g. participant and intervention characteristics, funding sources). Describe any assumptions made about any missing or unclear information.  **In a review investigating the cardiometabolic risk factor prevalence in South American children, the authors list and define all variables for which data were sought, including characteristics of the study design and participants:**  “Secondary outcomes were demographic data and study characteristics including age, rural vs. mixed vs. urban study setting, inclusion of indigenous population, year(s) of data collection, gender distribution, study location, study design, and study inclusion and exclusion criteria.” | Page 10  “Outcomes”  Lines 179-182 |
| Study risk of bias assessment | 11 | Specify the methods used to assess risk of bias in the included studies, including details of the tool(s) used, how many reviewers assessed each study and whether they worked independently, and if applicable, details of automation tools used in the process.  **In a review exploring the cardiometabolic risk factor prevalence in South American children, the authors specify the risk of bias tool used, the domains of bias addresses by the tool, how many reviewers assessed each study and how an overall judgement was reached:**  “Study quality was assessed using a modified Effective Public Healthcare Panacea Project Quality Assessment Tool for Quantitative Studies.(14) Studies were graded on selection bias and data collection methods, then graded as either “Strong,” “Moderate,” or “Weak” based on their pooled scores. Quality ratings for included titles are presented in Table 2.” | Page 10  “Study Quality”  Lines 184-187 |
| Effect measures | 12 | Specify for each outcome the effect measure(s) (e.g. risk ratio, mean difference) used in the synthesis or presentation of results.  **In a review examining the cardiometabolic risk factor burden in children in South America, the authors did not specify the effect measures as it was not appropriate for our review.** |  |
| Synthesis methods | 13a | Describe the processes used to decide which studies were eligible for each synthesis (e.g. tabulating the study intervention characteristics and comparing against the planned groups for each synthesis (item #5)).  **In a review quantifying the burden of cardiometabolic risk factors in children in South America, the authors did not report the process to decide which studies were eligible for each synthesis.** |  |
|  | 13b | Describe any methods required to prepare the data for presentation or synthesis, such as handling of missing summary statistics, or data conversions.  **In a review investigating the cardiometabolic risk factor prevalence in South American children, the authors report methods used to prepare and standardize the data for synthesis:**  “We performed descriptive statistics with Microsoft® Excel for Mac Version 16.54 and descriptive and analytical statistics with SPSS 28. Due to the risk of confounding variables, we performed a summative sub-analysis of median CMRF prevalence rates with same definitions using the filter features in SPSS. We used nonparametric, univariate, and multivariate testing. For variables with two groups we utilized the Independent-Samples Mann-Whitney U Test and for variables with more than 2 groups we used the Independent-Samples Kruskal-Wallis Test.” | Page 22  “Statistical Analysis”  Lines 197-202 |
|  | 13c | Describe any methods used to tabulate or visually display results of individual studies and syntheses.  **In a review exploring the cardiometabolic risk factor prevalence in South American children, the authors developed a summary table of study characteristics but did not present individual study results.**  See Table 2 | Pages 12-21 |
|  | 13d | Describe any methods used to synthesize results and provide a rationale for the choice(s). If meta-analysis was performed, describe the model(s), method(s) to identify the presence and extent of statistical heterogeneity, and software package(s) used.  **In a review quantifying the burden of cardiometabolic risk factors in children in South America, the authors report their chosen statistical analysis, along with rationale, and software packages used:**  ““We performed descriptive statistics with Microsoft® Excel for Mac Version 16.54 and descriptive and analytical statistics with SPSS 28. Due to the risk of confounding variables, we performed a summative sub-analysis of median CMRF prevalence rates with same definitions using the filter features in SPSS. We used nonparametric, univariate, and multivariate testing. For variables with two groups we utilized the Independent-Samples Mann-Whitney U Test and for variables with more than 2 groups we used the Independent-Samples Kruskal-Wallis Test.” | Page 22  “Statistical Analysis”  Lines 197-202 |
|  | 13e | Describe any methods used to explore possible causes of heterogeneity among study results (e.g. subgroup analysis, meta-regression).  **In a review investigating the cardiometabolic risk factor prevalence in South American children, the authors did not assess for causes of heterogeneity as this review does not compare outcomes.** |  |
|  | 13f | Describe any sensitivity analyses conducted to assess robustness of the synthesized results.  **In a review examining the cardiometabolic risk factor burden in children in South America, the authors report that conducting sensitivity analyses were inappropriate for the analysis.** |  |
| Reporting bias assessment | 14 | Describe any methods used to assess risk of bias due to missing results in a synthesis (arising from reporting biases).  **In a review quantifying the cardiometabolic risk factor prevalence in South American children, the authors report assessing selection and data collection bias with the EPPHPS tool.**  “Study quality was assessed using a modified Effective Public Healthcare Panacea Project Quality Assessment Tool for Quantitative Studies.(14) Studies were graded on selection bias and data collection methods, then graded as either “Strong,” “Moderate,” or “Weak” based on their pooled scores. Quality ratings for included titles are presented in Table 2.” | Page 10  “Study Quality Assessment”  Lines 184-187 |
| Certainty assessment | 15 | Describe any methods used to assess certainty (or confidence) in the body of evidence for an outcome.  **In a review exploring the cardiometabolic risk burden in South American children, the authors did not utilize methods to assess for certainty.** |  |
| **RESULTS** | | |  |
| Study selection | 16a | Describe the results of the search and selection process, from the number of records identified in the search to the number of studies included in the review, ideally using a flow diagram.  **In a review examining cardiometabolic risk factor prevalence in South American children, the authors report results of the search and selection process in text and in a flow diagram:**  “Figure 1 shows the PRISMA flow chart and screening process of all articles that the first search strategy yielded.” | Page 6  “Search Strategy”  Lines 156-157 |
|  | 16b | Cite studies that might appear to meet the inclusion criteria, but which were excluded, and explain why they were excluded.  **In a review investigating the cardiometabolic risk factor prevalence in South American children, the authors do not present a table with excluded studies, outside of the PRISMA flow diagram.**  See Figure 1 |  |
| Study characteristics | 17 | Cite each included study and present its characteristics.  **In a review quantifying the burden of cardiometabolic risk factors in children in South America, the authors include a table presenting for each included study the country, study design, setting, sample size, mean age, percentage of females, and outcomes assessed:**  See Table 3 | Pages 23-24 |
| Risk of bias in studies | 18 | Present assessments of risk of bias for each included study.  **In a review exploring cardiometabolic risk factor prevalence in South American children, the authors present a table indicating the domain-specific and overall risk of bias judgement for each study:**  See Table 2 | Pages 12-21 |
| Results of individual studies | 19 | For all outcomes, present, for each study: (a) summary statistics for each group (where appropriate) and (b) an effect estimate and its precision (e.g. confidence/credible interval), ideally using structured tables or plots.  **In a review investigating the burden of cardiometabolic risk factors in children in South America, the authors did not present summary statistics for each study included.** |  |
| Results of syntheses | 20a | For each synthesis, briefly summarise the characteristics and risk of bias among contributing studies.  **In a review examining cardiometabolic risk factor prevalence in South American children, the authors summarised various characteristics of the studies presenting individual prevalence rates of cardiometabolic risk factors in this population:**  See Table 3 |  |
|  | 20b | Present results of all statistical syntheses conducted. If meta-analysis was done, present for each the summary estimate and its precision (e.g. confidence/credible interval) and measures of statistical heterogeneity. If comparing groups, describe the direction of the effect.  **In a review quantifying the cardiometabolic risk factor prevalence in South American children, the authors report for a meta-analysis of individual cardiometabolic risk factor prevalence rates by median with interquartile range, comparison of subgroup analysis by country, setting and indigenous population:**  See Table 4 |  |
|  | 20c | Present results of all investigations of possible causes of heterogeneity among study results.  **In a review exploring the burden of cardiometabolic risk factors in children in South American, the authors present results of several subgroup analyses, indicating for each the p value for a test for subgroup differences:**  See Table 4 |  |
|  | 20d | Present results of all sensitivity analyses conducted to assess the robustness of the synthesized results.  **In a review investigating cardiometabolic risk factor prevalence in South American children, the authors did not conduct sensitivity analyses.** |  |
| Reporting biases | 21 | Present assessments of risk of bias due to missing results (arising from reporting biases) for each synthesis assessed.  **In a review examining burden of cardiometabolic risk factors in South American children, the authors did not explicitly assess for risk of bias due to missing results but did discuss data limitations in “Discussion” section.** |  |
| Certainty of evidence | 22 | Present assessments of certainty (or confidence) in the body of evidence for each outcome assessed.  **In a review quantifying the cardiometabolic risk factor prevalence in children in South American, the authors did not complete certainty assessments.** |  |
| **DISCUSSION** | | |  |
| Discussion | 23a | Provide a general interpretation of the results in the context of other evidence.  **In a review exploring cardiometabolic risk factor prevalence in South American children, the authors compare their findings with those in other relevant reviews:**  “Our study provides the most comprehensive assessment of CMRF in South American children. Krishnan and Short(194) assessed CMRF prevalence in South American children with type 1 Diabetes, but we did not find any other articles that focused on the general South American child and adolescent population. In comparison to other geographic populations, CMRF prevalence in South American children, and children in general, is poorly characterized. We could find few meta-analyses that focus on regional burdens of CMRF prevalence; many focus on a subset of the child and adolescent population, such as Krishnan and Short, or are concerned with assessing screening tools. Most regionally-focused literature on CMRF in children and adolescents details obesity and overweight prevalence, while dyslipidemia and impaired glucose metabolism are minimally characterized. The World Health Organization estimates that as of 2016, roughly 12.8% of children and adolescents aged 10-19 in the Americas experience obesity, the highest of all regions.(195) The next-closest region is the Western Pacific, with 7.4% of children experiencing obesity, which demonstrates how alarmingly high the prevalence of childhood and adolescent obesity is in the Americas as compared to the rest of the globe and the need to focus additional attention in this vulnerable region.” | Pages 31-32  “Comparison to Other Literature”  Lines 321-355 |
|  | 23b | Discuss any limitations of the evidence included in the review.  **In a review investigating the burden of cardiometabolic risk factors in children in South America, the authors describe various limitations in the included studies:**  “The greatest limitation in interpreting this data is the heterogeneity of definitions used for individual CMRF. As displayed in Table 2, there were 4-15 definitions used for each risk factor, which posed a difficulty in generating summary statistics from the data. We addressed this issue by generating additional summary statistics with data that shared the same cutoff definition. This issue would be avoided in future with international consensus on and wider acceptance of the use of standardized definitions for CMRFs in children. The absence of data from several South American countries limits the generalizability of this study to the whole region. Additionally, we extracted as much demographic information as possible; however, we were unable to extract basic demographic data such as gender for each definition, as many studies reported more than one risk factor but not necessarily gender distribution or ages per risk factor. Therefore, we do not know if CMRF are clustered further in one gender over another or one age group over another. A further limitation was the minimal indication of the inclusion of indigenous populations; although many South American populations include indigenous members, only a handful of studies specifically noted the inclusion of majority indigenous groups. This likely underestimates the prevalence of CMRF in indigenous individuals, and there was limited data on which to assess variation in subgroups such as country.” | Page 32  “Limitations”  Lines 337-352 |
|  | 23c | Discuss any limitations of the review processes used.  **In a review examining cardiometabolic risk factor prevalence in South American children, the authors report limitations of the review process used:**  “When compiling the titles from the basic Google Scholar search, there was a server error after uploading 980 titles from this database despite multiple attempts. Therefore, only 980 of the 1,060 titles from this search were able to be included for screening. Titles from Google Scholar were imported using the Mendeley plug-in for Google Chrome. This software did not differentiate between Spanish and English versions of the same text, so there were many duplicate titles that were imported and had to be screened out, leading to a much higher initial number of titles than the hits found by the searches.” | Page 33  “Limitations”  Lines 353-359 |
|  | 23d | Discuss implications of the results for practice, policy, and future research.  **In a review quantifying the cardiometabolic risk factor prevalence in South American children, the authors discuss the implications for policy and future research:**  “Overall, children in rural settings and indigenous children trended to lower prevalence rates of CMRF, which is unexpected as many studies on the nutritional status of indigenous populations worldwide suggest a growing double burden of undernutrition and obesity in indigenous children.(197) This inconsistency could be attributed to the small sample sizes and lack of available data on most of the diverse indigenous populations in South America. Brazil, Chile, Colombia, and Argentina were the most represented in terms of number of studies. Though Peru had the largest population, this is due to one study (Torres-Roman et al.) including a population of over 2 million. In terms of CMRF prevalence, from our analysis Chilean children have the highest prevalence rates of all CMRF except HBP. This is a noteworthy finding and indicates that Chilean children may be more at risk for developing metabolic syndrome as adults and experiencing long term health repercussions than their peers in other South American countries.  Many South American countries were underrepresented or not represented at all in our analysis. There were three or fewer studies that contained data from Bolivia, Paraguay, and Uruguay for each CMRF, and many countries only had data reported on obesity. Many countries, including French Guiana and Suriname, did not have any available or relevant articles. Indigenous populations were vastly underrepresented in the analysis, with only 8 studies reporting data specifically about indigenous populations, and most only reporting on obesity, and all from rural settings. Almost all the studies on indigenous populations were completed by Hirschler et al. in Argentina. As indigenous populations are present in practically every South American country, it is important to capture the CMRF prevalence in all countries to assess the future health and burden of CMRF in these groups.” | Pages 33-34  Lines 373-395 |
| **OTHER INFORMATION** | | |  |
| Registration and protocol | 24a | Provide registration information for the review, including register name and registration number, or state that the review was not registered.  **In a review exploring the burden of cardiometabolic risk factors in children in South American, the authors report that this review was not registered.** |  |
|  | 24b | Indicate where the review protocol can be accessed, or state that a protocol was not prepared.  **In a review quantifying the cardiometabolic risk factor prevalence in South American children, the authors state that a protocol was not prepared.** |  |
|  | 24c | Describe and explain any amendments to information provided at registration or in the protocol. |  |
| Support | 25 | Describe sources of financial or non-financial support for the review, and the role of the funders or sponsors in the review.  **There were no sources of funding for this review.** |  |
| Competing interests | 26 | Declare any competing interests of review authors.  **The authors declare no conflicts of interest.** |  |
| Availability of data, code and other materials | 27 | Report which of the following are publicly available and where they can be found: template data collection forms; data extracted from included studies; data used for all analyses; analytic code; any other materials used in the review.  **Data used for all analyses are publicly available and can be found using our search strategy outlined in Table 1. Data collection forms are not publicly available but are available upon request.** |  |

*From:*  Page MJ, McKenzie JE, Bossuyt PM, Boutron I, Hoffmann TC, Mulrow CD, et al. The PRISMA 2020 statement: an updated guideline for reporting systematic reviews. BMJ 2021;372:n71. doi: 10.1136/bmj.n71

For more information, visit: <http://www.prisma-statement.org/>
