# Supplementary material for: Cardiometabolic risk factors in South American children: A systematic review and meta-analysis
Source: PLoS One. 2023 Nov 22;18(11):e0293865. doi: 10.1371/journal.pone.0293865 (PMC10664905; doi:10.1371/journal.pone.0293865)
Supplement: Attachment — Submitted filename: Review Cardiometabolic Risk Factors in South American Children.docx [file pone.0293865.s002.docx]

**Review**

I reviewed the manuscript entitled “Cardiometabolic Risk Factors in South American Children: A Systematic Review and Meta-Analysis”. This is an interesting article assessing the effect of WWI on stroke risk. The manuscript is in the scope of the journal, but certain shortcomings should be addressed before the article could be published:

•	Several language and grammar editing is required.

•	The introduction section is too long. Some information mentioned here belongs to the discussion section

•	Why did you exclude Caribbean islands from geographical region of South America? Moreover, as mentioned in the article the data from several South American countries data are missing, how does this affect the generalizability of the results to South America.

•	In the abstract you have mentioned 1,540 screened studies, however, in the results (Line 206) “2,181 studies screened” is written. Which one is the correct number?

•	Figure 2 is missing in the manuscript.

•	As you already mentioned in the limitation, heterogeneity of definitions of CMRF is the main concern. How have you addressed that. Please elaborate

- Is the inter-study heterogeneity studied?
