# Supplementary material for: Cardiometabolic risk factors in South American children: A systematic review and meta-analysis
Source: PLoS One. 2023 Nov 22;18(11):e0293865. doi: 10.1371/journal.pone.0293865 (PMC10664905; doi:10.1371/journal.pone.0293865)
Supplement: Attachment — Submitted filename: CMRF.Haugh.Response to Reviewers.docx [file pone.0293865.s003.docx]

PLOS One Response to Reviewers for “Cardiometabolic Risk Factors in South American Children: A systematic review and Meta-Analysis”

Thank you for your constructive comments and the time you took to review our work. Please find our responses below. Please note that the line references are given as lines in the tracked changes version/ lines in clean version.

1. We have reviewed the document for language and grammar edits.
2. We made several edits to the Introduction section, as detailed in the revised manuscript. Notably, we moved two paragraphs about the utility of metabolic syndrome in children to the Discussion section.
3. We excluded the Caribbean Islands due to their increased cultural and culinary heterogeneity as compared to the countries conventionally considered South America. We have addressed this in lines 136-137/112-113. In results and discussion (lines 212-214/187-188 and 372-373/340-341 respectively), we list the South American countries and territories that did not have data. We discussed this as a limitation to generalizability in our Discussion section (lines 373-374 / 341-342).
4. The number originally listed in the abstract was the total number of articles screened before we added the additional, targeted Google searches. This number has been updated to match the number in the body of the article.
5. Figures 1 and 2 are in a separate document per editor’s instructions. Please let the editor know if you are unable to view this document.
6. We address heterogeneity in our analysis by completing separate statistical analysis by the most used definition of each CMRF to assess the effect of the differing definitions on our outcomes (addressed in Methods 201-203/175-177 and Discussion in lines 366-371/335-336. We did not find a statistical test that would be appropriate to test the heterogeneity of our studies.
